# Multi-Modal FHIR Sensor Fusion for Real-Time Clinical Decision Support: An Architecture for the Hospital Bedside and Its Extension Toward Remote Patient Monitoring

**DOI:** 10.3390/s26217026

**Published:** 2026-11-06

**Authors:** Pierre Boulanger

**Affiliations:** 1 Department of Computing Science, University of Alberta, Edmonton, AB T6G 2E8, Canada; pierreb@ualberta.ca; 2 Department of Radiology and Diagnostic Imaging, University of Alberta, Edmonton, AB T6G 2B7, Canada

**Keywords:** sensor fusion, telemedicine, remote patient monitoring, FHIR R4, real-time streaming, clinical decision support, wearable sensors, ensemble learning, explainable AI, multi-modal data integration

## Abstract

MedROAD V3 is a real-time FHIR R4 streaming platform that turns clinical sensor streams into calibrated deterioration risk, evaluated retrospectively on a cardiac ICU cohort. FHIR event sourcing, Kafka and Faust assemble an 86-element feature vector every five minutes. An XGBoost, LSTM and Transformer ensemble, combined by logistic meta-learning with SHAP attribution, scores each window; a write-back client returns the alert to the chart with a runtime-filtered language-model narrative. On 5049 held-out MIMIC-IV patients it reaches AUROC 0.816 at an Expected Calibration Error of 0.001, with a median inference path of 3.1 ms, so latency is governed by the EHR rather than by computation. The FHIR Observation resource is device-agnostic, so the pipeline ingests wearable streams unmodified. Three limits qualify these results. It is retrospective, and the remote-monitoring figures are a feature-restriction analysis on ICU data, not a wearable validation. Twenty-nine columns recording only how a patient was documented and managed, none physiological, reach AUROC 0.767 on the same split, so much of the discrimination reflects care process rather than physiology, although removing them costs only 0.007. Because OpenEMR 7.0.2 reference server grants no clinical FHIR write scope, the loop closes over an alternative path using its REST API, writing the alert as a Subjective-Objective-Assessment-Plan (SOAP) note on the encounter. The substitution works but is vendor-specific rather than portable, so the closed loop shown here is conditional on the target server.

## 1. Introduction

The intensive care unit (ICU) represents one of the most data-dense environments in modern medicine. A patient in the single cardiac ICU can generate hundreds of physiological measurements per hour through bedside monitoring systems, laboratory analysers, ventilators, and injection pumps. Despite this abundance of data, clinically actionable insight often lags behind physiological reality by hours—a direct consequence of batch-processing architectures in which electronic health record (EHR) data are periodically extracted, analysed, and reviewed rather than continuously monitored.

Early warning score systems such as the National Early Warning Score (NEWS) [1] have demonstrated that continuous physiological monitoring and timely escalation can significantly reduce hospital mortality. However, rule-based threshold systems suffer from poor specificity, generating alert rates that overwhelm nursing and physician workflows and produce the well-documented phenomenon of alert fatigue [2,3]. Machine learning approaches have achieved superior discriminative performance over rule-based scores in predicting deterioration [4,5], but their integration into real-time clinical workflows, closing the loop between prediction and physician notification, remains an open engineering and informatics challenge.

The HL7 FHIR R4 standard [6,7] has emerged as the dominant framework for clinical data exchange, with OpenEMR representing a widely deployed open-source EHR with native support for FHIR R4 (openemr.org). FHIR’s Subscription mechanism provides a REST-hook push channel that transforms a passive FHIR server into an event stream source, yet the informatics literature contains limited examples of this mechanism being exploited for real-time ML-driven decision support with bidirectional write-back.

The same architectural gap is acutely felt in telemedicine and remote patient monitoring (RPM). The COVID-19 pandemic accelerated the deployment of home monitoring devices: pulse oximeters, continuous glucose monitors (CGM), portable single-lead ECG patches and Bluetooth-enabled blood pressure cuffs—generating continuous multi-modal physiological streams that clinicians must interpret without the dense contextual scaffolding of the hospital environment. Post-discharge heart failure patients monitored at home, for example, can transmit weight, SpO_2_ and resting heart rate observations daily, yet the clinical decision workflow for acting on these streams accurately mirrors the ICU problem: latency between observation and alert is clinically costly, rule-based thresholds generate excessive false positives, and the narrative context required for physician action is absent. The FHIR R4 Observation resource is the standardised container for both bedside monitor readings and wearable device output, and an architecture designed around native FHIR streaming therefore inherits sensor-agnosticism by construction.

The sensing side of this problem is progressing faster than the informatics side. Wearable optical and photonic transducers now recover cardiorespiratory waveforms from substrates that were until recently the province of laboratory instrumentation, and reviews of fibre-based wearable sensing document a steady move from single-parameter prototypes towards multi-modal physiological capture [8]. Devices in this class are increasingly multi-purpose: a recent smartphone-based photonic mHealth system recovers plantar pressure distribution together with cardiac and respiratory activity from one instrument, performing the inference on the phone itself [9]. The direction of travel matters for the present work because it sharpens rather than relieves the integration problem. A single device that emits several physiological channels at heterogeneous rates, with on-device inference producing derived quantities alongside raw measurements, is exactly the input an architecture organised around a device-specific ingestion path handles badly. Treat every channel as an FHIR Observation, whatever product it produced, is what allows such a device to be added without touching the pipeline downstream of ingestion. We return to this in Section 3.6, where the limits of the claim are set: the present feature vector does not have an element for several channels published by these devices, so sensor-agnostic ingestion is not the same as sensor-agnostic prediction.

This paper describes MedROAD V3, a direct successor to the original MedROAD system [10]. The published MedROAD V2 is an AI-integrated electronic medical record in its own right, pairing a four-model ensemble (gradient boost, isolation forest, autoencoder and LSTM) over a 72-dimensional feature vector with SHAP explanations and a Claude API assistant, and surfacing results through its own clinical dashboard and mobile interface. MedROAD V3 keeps the ensemble-plus-SHAP plus-narrative design and changes where it runs: inference is driven by individual FHIR resource arrivals rather than by 15-min resampling, the feature vector is extended from 72 to 86 elements, a full calibration and online recalibration pipeline is added, and outputs are written back into a third-party EHR rather than presented in the system’s own interface. Section What V3 Changes, and What It Does Not outlines that relationship accurately, including what V3 does not improve on.

This paper makes the following new contributions beyond the published MedROAD V2 [10]:

1. A complete real-time FHIR streaming architecture replacing the published MedROAD V2 batch ETL path, with FHIR Subscription-driven event sourcing, Apache Kafka message brokering and Faust windowed stream processing.
2. An expanded 86-element feature vector (up from 72 in the published MedROAD V2 [10]), adding serial delta features for troponin I, NT-proBNP and lactate; two physiological interaction scores (SIG, BCR); and five new metadata features, bringing the metadata block from three to eight, encoding documentation timing, presence of the ABG panel, IV drip status, and  length of stay in the ICU.
3. A complete model calibration pipeline—Platt scaling (XGBoost), temperature scaling (LSTM, Transformer), and isotonic regression (meta-learner)—with online site-specific recalibration triggered by rolling calibration drift detection.
4. A bidirectional FHIR R4 write-back client that emits alerts as native Flag, CommunicationRequest, DocumentReference, and ServiceRequest resources, together with a second, vendor-specific path over OpenEMR’s standard REST API that emits the alert and its narrative as a SOAP note on the encounter. The FHIR path could not be exercised against the OpenEMR 7.0.2 reference deployment, which does not offer write scope for any FHIR clinical resource; the standard-API path runs there, so the loop closes at the cost of portability (Section 11.4).
5. An extended Claude API integration with a structured prompt architecture, a runtime factual-grounding filter, and output-contract checks that divert a failing narrative to a deterministic fallback template, structural immunity to prompt injection, and pseudonymisation of the patient identifier before transmission. The component has been implemented but not clinically validated: no prospective evaluation of narrative quality has been conducted (Section 7.9).
6. End-to-end latency characterisation, and a preliminary regulatory analysis under the Health Canada SaMD framework offered as the authors’ interpretation rather than as a determined classification.

The remainder of this paper is organised as follows. Section 2 surveys the four literatures that this work draws together—FHIR interoperability, stream processing architectures, machine learning for deterioration prediction, and explanation and alert fatigue in clinical decision support—and states which limitations of the published MedROAD architecture the present work addresses. Section 3 describes the five functional layers and closes with the remote and wearable sensor case. Section 4 derives the 86-element feature vector from its six sub-vectors and specifies the imputation strategy. Section 5 describes the three base learners, the meta-learner, the SHAP computation, and the derivation of the alert threshold. Section 6 treats probability calibration as a first-class concern: the metrics, each base model individually, distributional shift and site-specific recalibration, and the effect of class imbalance. Section 7 covers the language model layer, from the mitigation of prompt contract and hallucinations to the deterministic fallback and a working example of the generated output. Section 8 addresses write-back and OpenEMR integration. Section 9 describes the cohort and protocol, decomposes the measured latency by stage, and compares the ensemble against NEWS and MEWS at a matched sensitivity. Section 10 places the system within the Health Canada software-as-a-medical-device framework. Section 11 discusses remote monitoring applications, closed-loop against dashboard architectures, and the limitations of this work. Section 12 concludes.

## 2. Related Work

### 2.1. Interoperability, Decision Support, and Streaming

HL7 FHIR has become the dominant standard for clinical data exchange [7], with SMART-on-FHIR establishing the application layer above it [11] and subsequent work documenting both its adoption trajectory and its persistent implementation heterogeneity [12,13]. What this literature does not address is the use of FHIR Subscriptions as a real-time event source for machine learning.

The determinants of clinical decision support efficacy are now well characterised. Kawamoto et al. [14] identified automatic provision within workflow, delivery at the point of decision, and actionable recommendations as the features that separate effective systems from ineffective ones, findings reinforced by later systematic reviews [15,16,17]. MedROAD V3 is designed around these determinants rather than around model performance alone.

On the infrastructure side, Kafka [18] and the stream processing semantics formalised by Flink [19] and the Dataflow model [20] provide the durability and windowing guarantees required for the processing of ordered events per-patient. These are mature technologies in industrial settings, but remain rare in clinical informatics deployments.

### 2.2. Prediction, Explanation, and Their Clinical Reception

Machine learning for predicting deterioration is well established. Multicenter comparisons found gradient enhancement to outperform logistic regression and rule-based early warning scores [4], large deployments have demonstrated operational feasibility [5,21], and deep models have achieved strong performance on acute kidney injury and general hospital outcome on scale [22,23].

The three learners combined here are each individually established. XGBoost [24], based on gradient boosting [25], handles missingness natively and has been applied to the prediction of septic shocks while maintaining interpretability through the importance of features [26]. LSTM networks [27] learn clinically meaningful representations from multivariate ICU series, outperforming hand-crafted features [28], and standardised MIMIC-III benchmarks established that multitask learning consistently beats single-task models [29]. GRU-D addressed irregular sampling with a learnable decay toward the empirical mean [30], motivating the explicit missingness features used here as a lighter proxy. Following the Transformer [31], self-attention has been adapted to structured EHR data [32] and to irregularly sampled observations treated as set elements rather than imputed series [33], the last particularly relevant to asynchronous streaming.

Explainability has converged on SHAP for its uniqueness guarantees and exact polynomial-time computation on tree ensembles [34,35]. Its clinical reception is more contested. Interviews with intensivists found that clinicians want to know which factors drive a prediction and how it would change under intervention [36], while others caution that post-hoc explanations can create unwarranted confidence [37,38] or argue that high-stakes decisions warrant inherently interpretable models instead [39]. MedROAD V3 adopts SHAP while treating calibration, not explanation, as the primary trust mechanism.

The same tension appears with language models. Strong medical benchmark performance [40,41,42] coexists with the documented propagation of race-based misconceptions [43], which is why narrative generation here is constrained to summarising a calibrated score and its attribution rather than performing a diagnosis [10].

Alert fatigue is the failure mode these systems most often encounter in practice: clinicians override between roughly half and 96% of medication safety alerts [3,44], and levels of alerts by severity, so that only the most serious interruption to the prescriber raises compliance [45]. Conditioning narrative generation on a calibrated threshold is MedROAD V3’s response to this.

### 2.3. Cardiac Applications, Federation, and Regulation

In cardiology specifically, deep models have matched or exceeded cardiologist performance in single-lead ECG arrhythmia detection [46] and have been surveyed in risk prediction and imaging tasks [47,48,49].

Federated learning offers a route to multi-site training without data movement [50,51], demonstrated at scale for COVID-19 outcome prediction in 20 institutions [52], though statistical heterogeneity between sites remains an open problem [53] and reproducibility in clinical ML is itself inconsistent [54]. The site-specific recalibration procedure of Section 6.3 is a lighter alternative that addresses the same heterogeneity.

Regulatory frameworks treat software of this kind as a medical device [55,56]. Of the hundreds of AI-based devices cleared to date, few are continuously learning and most were authorised through pathways that require only substantial equivalence [57], with the transparency regarding training data and validation remaining limited [58].

### 2.4. Published MedROAD V2: Prior Architecture and Limitations

The published MedROAD V2 [10] is an AI-integrated electronic medical record system in its own right, rather than an analytics layer attached to a third-party electronic health record (EHR). It comprises a patient management system, a physiological analytics engine, a laboratory information system interface that supports HL7 v2.5.1 and FHIR R4, a Claude API assistant generating explainable interpretations grounded in the literature, and a presentation layer of clinical dashboards, configurable alert management, and a responsive mobile web interface.

Its analytics engine is an ensemble of four complementary models: gradient boost for supervised prediction, isolation forests for anomaly detection, autoencoders for pattern recognition, and an LSTM for temporal modelling. Raw vital signs and laboratory values are resampled at 15-min intervals over a 24-h history and transformed into a 72-dimensional feature vector of statistical summaries, temporal trends, variability metrics, and cross-parameter-derived quantities such as the shock index. The explanations use TreeSHAP for the gradient-boosting component and DeepSHAP for the neural components. On MIMIC-IV v2.2, over 38,426 ICU admissions from 31,847 patients under a temporal split, V2 reports AUROC 0.87 (95% CI 0.85–0.89) for composite 12-h deterioration at a 12.6% admission-level event rate, with AUC-PR 0.56 and a positive predictive value of 0.42, against NEWS2 at AUROC 0.72 and SOFA at 0.76 on the same cohort. V2’s latency figures are per-stage architectural estimates rather than measurements, and allow 60 ms for ensemble inference and 5 ms for SHAP attribution, with predictions updated within 30 s of new data arrival.

#### What V3 Changes, and What It Does Not

Because the novelty of the present work is framed in relation to V2, the relation is precisely stated. V3 is not a better model than V2 and does not claim to be. The two solve different prediction problems and their headline figures are not comparable. V2 predicts the level of admission to the ICU in a general adult ICU population; V3 predicts at the level of a five-minute window in a cardiac ICU cohort selected for instability, where the window-level event rate is 4.4% rather than 12.6% at the admission level. Window-level prediction in a pre-selected unstable population is the harder task, and V3 additionally enforces patient-grouped splits, which remove a source of leakage that a temporal split by admission year does not fully exclude. The cohorts differ (all adult ICU against CCU and CVICU), the MIMIC-IV versions differ (v2.2 against v3.1) and the evaluation units differ. Comparing 0.87 with 0.816 would be comparing two different measurements.

What V3 contributes is architectural, and the three differences are real. The first is the integration target: V2 surfaces its outputs in its own dashboard, because V2 is the record system; V3 writes to a third-party EHR over FHIR, which is the harder problem and the one that a site with an incumbent EHR actually faces. The second is the granularity of the event: V2 resamples to 15-min intervals over a 24-h history, while V3 processes five-minute tumbling windows driven by the arrival of individual FHIR resources, and reduces the inference path from the 65 ms V2 allows for inference and attribution together to 3.1 ms measured at the median. The third is calibration: V2 reports discrimination and an operating point but without probability calibration, whereas V3 adds Platt, temperature, and isotonic stages with site-specific recalibration, which is what makes the risk score interpretable as a probability and transferable between sites.

In that comparison, three properties of V2 are taken into account and are easily mistated. V2 has no five-minute batch ETL latency floor; it reports updating predictions within 30 s of new data arrival. Its presentation layer is a web dashboard and mobile interface, not a desktop application that requires clinicians to leave another EHR. And OpenEMR plays no role in V2, whose alerts are written in its own record and delivered through dashboards, pagers, secure messaging, and EHR in-basket notifications over FHIR endpoints.

MedROAD V3 addresses the integration, granularity, and calibration differences set out above. The batch ETL path is replaced by FHIR event streaming, Subscription-driven where the server supports it and polled otherwise, reducing the system’s measured computation to a few milliseconds; the one internal stage still estimated rather than measured, the Kafka consumer poll, is given in Section 9.4. Inference outputs are written back to the patient chart, as native FHIR resources, surfaced within the clinician’s existing session without context switching. The vector of features is expanded from 72 to 86 elements, adding temporal delta features, physiological interaction scores, and the eight metadata features described in Section 4.4. A complete probabilistic calibration pipeline is added (Section 6), which was absent from the published MedROAD V2. Table 1 summarises the principal architectural differences.

## 3. System Architecture

**Figure 1:**
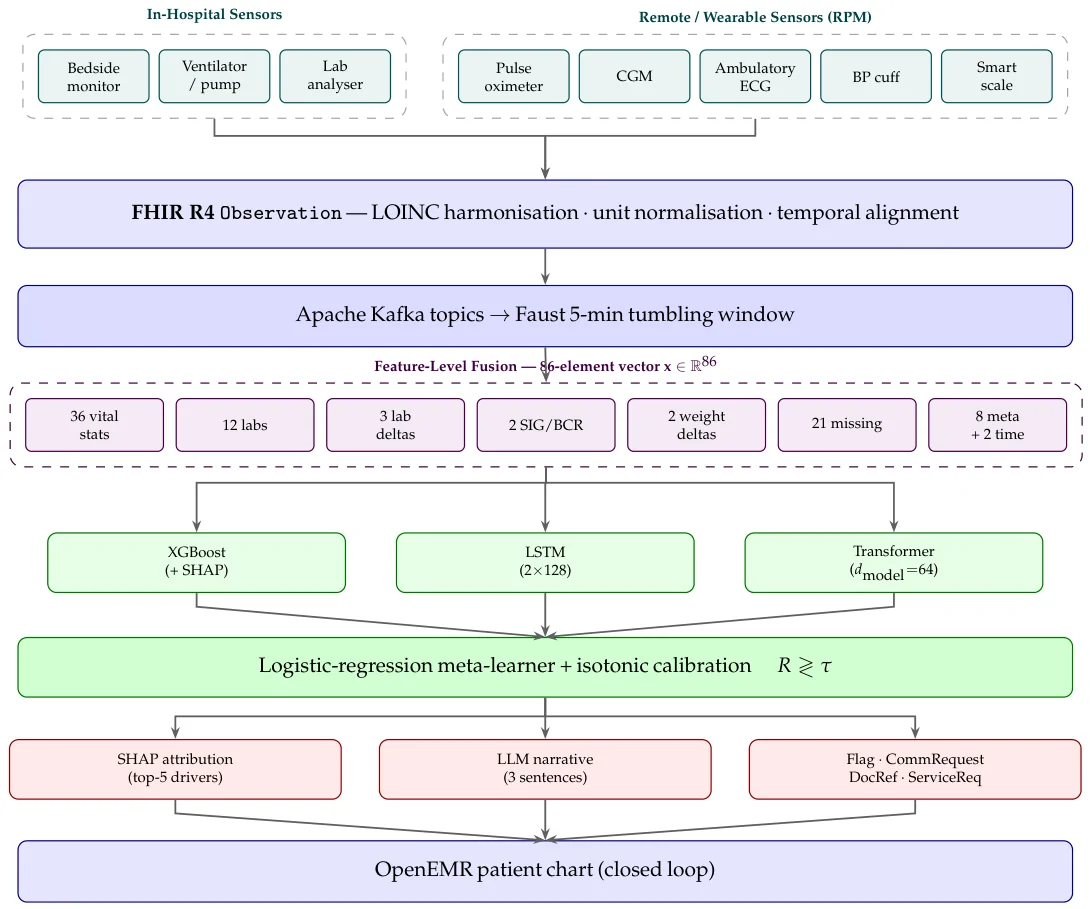
Multi-modal sensor fusion topology in MedROAD V3. Heterogeneous in-hospital and remote sensor classes (top) are normalised to a common FHIR R4 observation representation, which makes the downstream pipeline device-agnostic: data-level fusion. Windowed streams are assembled into a single 86-element vector, shown here as the seven blocks the feature builder emits and grouped in the text into six sub-vectors: feature-level fusion. Three heterogeneous learners are combined by a calibrated meta-learner: decision-level fusion. Outputs are written back to the patient chart, as native FHIR resources where the server permits and over the server’s own REST API where it does not, closing the loop.

MedROAD V3 is organised into five functional layers, as shown in Figure 1. Each layer is independently deployable and communicates with adjacent layers via well-defined interfaces: FHIR REST for EHR interaction, Kafka topics for event streaming, and HTTP/JSON for ML inference calls.

Before describing each layer in detail, it is useful to characterise the system in sensor fusion terms, because the architectural choices that make MedROAD V3 device-agnostic are precisely those that determine at which level heterogeneous sensor evidence is combined. Figure 1 maps the pipeline to the conventional three-level fusion taxonomy. Data-level fusion occurs at the FHIR R4 observation boundary, where readings from bedside monitors, laboratory analysers, and consumer wearables are harmonised into a single LOINC-coded representation with normalised units and aligned timestamps; downstream components never observe the originating device class. Feature-level fusion occurs in the Faust stream processor, where windowed observations from all modalities are assembled into one 86-element vector, drawn here as the seven blocks the builder emits (Section 4). Decision-level fusion occurs in the ensemble, where three learners with complementary inductive biases—gradient-boosted trees over tabular features, a recurrent model over temporal sequences, and a self-attention model over irregular intervals—are combined by a calibrated meta-learner (Section 5). This separation is what allows the wearable extension of Section 3.6 to require only a change of feature mapping, rather than a change of architecture.

### 3.1. FHIR Event Source Layer

The event source layer admits two implementations, and which applies is a property of the FHIR server rather than of MedROAD. Where the server implements Subscription, five Subscriptions are registered at system initialisation via an idempotent bootstrap script, using the R4B criteria syntax targeting the types and categories of resources listed in Table 2; matching resources are then pushed to the webhook receiver as they are written.

Subscription is an optional part of FHIR R4 and is not universally implemented. OpenEMR 7.0.2 is one such server: its CapabilityStatement advertises 27 resource types, and Subscription is not among them, so a REST-hook Subscription cannot be registered against it and the push path is unavailable. For such servers, MedROAD polls the same resource types on a fixed interval, using _lastUpdated as a high-water mark, and emits anything new onto the same Kafka topics. Everything downstream of the event source—windowing, feature assembly, inference, and write-back—is identical on either path, and MedROAD selects between them by reading the server’s CapabilityStatement at startup rather than by configuration.

The two are not equivalent, and the difference belongs in any latency claim. Push latency is bounded by delivery; poll latency is bounded below by the poll interval, so a 30-s interval dominates the millisecond-scale computation path of Section 9.4 by three orders of magnitude. Polling also misses a written and reverted change between passes, and its query load scales with the number of monitored resource types rather than with the event rate. Where Subscription is available, it is strictly preferable; the polling path exists so that the architecture is deployable against the EHR this work targets, not as an equal alternative.

Each subscription delivers the full FHIR resource payload to the MedROAD ingestion endpoint through a REST-hook channel secured by an HMAC-SHA256 signature (header: X-MedROAD-Signature). Signature verification prevents spoofed clinical events from entering the Kafka pipeline.

### 3.2. Message Broker Layer

Apache Kafka provides three properties critical to clinical deployment:

Durability. All FHIR events persist on disc with configurable retention (default 30 days), allowing complete replay for audit, retrospective analysis, or model retraining [59].

Fan-out. The LSTM inference consumer, audit log consumer, and alert rule engine operate as independent consumer groups reading from the same topics, enabling modular system evolution without upstream changes.

Backpressure. Kafka decouples production (FHIR events from OpenEMR) from consumption (Faust inference), preventing cascade failures under high patient census.

The topic partition is keyed to patient_id, ensuring that all events for a given patient are consumed in order by the same Faust stream processor instance, a requirement for correct temporal windowing [20]. The reference deployment uses a 3-broker Apache Kafka cluster. For single-server deployments serving 20–50 concurrent patients, NATS JetStream offers equivalent at-least-once delivery semantics with lower operational overhead.

### 3.3. Stream Processing Layer

The Faust stream processor maintains a five-minute tumbling window of vital-signal samples per patient using persistent tables backed by RocksDB. For each observation of the incoming vital-sign, the processor (1) updates the window, (2) performs a 24-h look-back join with cached laboratory values, (3) constructs the 86-element feature vector described in Section 4, and (4) sends it to the inference engine. The window readiness is gated on a minimum of three different vital-sign LOINC codes, preventing spurious inference on newly admitted patients with incomplete physiological profiles.

### 3.4. Inference and Decision Layer

The inference pipeline is described in Section 5. In particular, the Claude API is invoked only when the ensemble risk score *R* exceeds a configurable threshold τ, derived from the $F_{1}$-maximising operating point in local held-out data (Section 7.7). This conditional invocation architecture—rather than narrative generation in every window—is the primary mechanism to reduce alert fatigue.

### 3.5. Write-Back Layer

Inference results are returned to the patient chart so that they are visible in the clinician’s existing EHR workflow rather than in a separate interface. Two interfaces are implemented, and which one is applied is a property of the target server. Where a server supports FHIR writes, results are written as native FHIR resources, which are portable and carry structured provenance. OpenEMR 7.0.2 offers no write scope for any FHIR clinical resource, so on the reference deployment the write-back instead goes over OpenEMR’s own REST API, emitting the alert and its narrative as a SOAP note on the encounter. Section 8 describes both and the trade-off between them.

### 3.6. Extension to Remote Patient Monitoring Sensor Streams

A key architectural property of MedROAD V3 is that the FHIR event source layer is sensor-agnostic. The Kafka ingestion webhook accepts any valid FHIR R4 Observation resource regardless of its originating device. The type of resource, not the device class, determines the assignment of Kafka topics (Table 2). This means that wearable and home monitoring devices that publish FHIR R4 Observations—a growing category that includes certified Bluetooth pulse oximeters, CGMs, ECG patches, and blood pressure monitors supported by Apple Health, Google Fit, and Withings Health Integration Hub—can be routed through the identical Kafka-Faust pipeline without any modification of the broker, stream processor, or inference engine.

The primary adaptation required for RPM deployment is the reconfiguration of the feature vector. The current 86-element vector (Section 4) is calibrated for the cardiac ICU setting: it assumes five-minute observation windows with dense coverage of vital-signs. Remote monitoring streams are typically sparser—a CGM produces a reading every five minutes, a resting ECG patch may produce a daily summary, and weight observations arrive once per day–requiring window widths and imputation strategies appropriate to the observation frequency of each device class. Table 3 maps the ICU monitoring channels to their remote-monitoring analogues. Channels without a wearable counterpart, such as arterial-line MAP and ventilator peak pressure, would be replaced by candidate RPM-specific features—activity energy expenditure, step cadence, sleep apnea index—derived from consumer-grade wearables.

To quantify what this cost, MIMIC-IV windows were degraded to what each kit could actually observe: channels without wearable analogue were removed, observable channels were coarsened to their device cadence, the window was widened to 24 h, and the scorer was recalibrated on the degraded representation. Table 4 reports the result.

Discarding 47 of 86 features and widening the window by a factor of 288 costs 0.034 AUROC. Most of the deterioration signal available to the full ICU feature set survives restriction to channels that a three-device home kit can measure. The reference row, 0.759, agrees with the logistic baseline of Table 5 at 0.758 in the same cohort, which is the consistency check that the two experiments score the same thing.

Four caveats bound this result and together set what it may be taken to show.

No wearable data were used. So, the experiment degrades the MIMIC-IV ICU data to the channels observed by a home kit. It does not evaluate the wearable sensor streams, which this work has not done. The mapping of features RPM in Table 3 is a design proposal derived from the FHIR specification and clinical reasoning, not an empirical result.

Sparsity only. degradation simulates reduced channel coverage and a wider window. Consumer devices add measurement error, motion artefact and adherence gaps on top, so these figures are an upper bound on what a real kit would achieve.

A different scorer. The degradation deliberately uses logistic regression rather than the ensemble, so that the measured loss reflects the restriction of features rather than differences in model capacity. The reference figure of 0.759 is therefore not comparable to 0.816 reported elsewhere, and −0.034 should be read as the cost of feature restriction under a fixed simple scorer, not as the degradation the deployed ensemble would suffer. Repeating it with the ensemble is straightforward and is a subject for future work.

Two of the five devices contribute nothing. the five device kit performs identically to the three-device kit because the continuous glucose monitor and the ambulatory ECG publish channels, interstitial glucose and cardiac rhythm, for which the feature vector does not have an element. The identical rows in Table 4 are therefore a statement about the feature vector rather than about the devices, and adding hardware cannot help until the vector is extended.

Architectural compatibility is not validated RPM performance, and the reference in the title to remote patient monitoring should be read as describing the reach of the architecture rather than a demonstrated capability. Latency is not the binding constraint in this setting: the ICU figures in Section 9.4 are the worst case for asynchronous wearable streams, and against the hour-to-day decision timescale of chronic disease management even the 1.8 s narrative call is irrelevant.

## 4. Feature Engineering

The MedROAD V3 feature vector contains D=86 elements structured as follows, expanded from the 72-element vector used in the published MedROAD V2 [10]:

(1) $$x=[x_{\mathrm{vital}},\;x_{\mathrm{lab}},\;x_{\mathrm{weight}},\;x_{\mathrm{miss}},\;x_{\mathrm{time}},\;x_{\mathrm{meta}}]$$

where $x_{\mathrm{vital}}\in R^{36}$ (nine parameters × four statistics), $x_{\mathrm{lab}}\in R^{17}$ (twelve values, three serial deltas, two interaction scores), $x_{\mathrm{weight}}\in R^{2}$, $x_{\mathrm{miss}}\in R^{21}$ (per-channel availability indicators), $x_{\mathrm{time}}\in R^{2}$ and $x_{\mathrm{meta}}\in R^{8}$.

### 4.1. Vital Sign Features ($x_{\mathrm{vital}}$)

Nine vital-sign parameters are monitored according to the LOINC codes in Table 6. For each parameter $v_{k}$, four statistics are calculated in the five-minute window W=[t−300,t]: the mean $\mu_{k}$, the standard deviation $\sigma_{k}$, the minimum $m_{k}$ and the rate of change ${\dot{v}}_{k}=\left(v_{k}^{\left(\mathrm{last}\right)}-v_{k}^{\left(\mathrm{first}\right)}\right)/\left(t_{\mathrm{last}}-t_{\mathrm{first}}\right)$, giving $|x_{\mathrm{vital}}|=9\times 4=36$. The minimum is retained in preference to the maximum because deterioration in cardiac patients usually manifests itself as transient minima, such as episodes of SpO_2_ desaturation, rather than as shifts in the mean of the window.

### 4.2. Body Weight and Its Trend ($x_{\mathrm{weight}}$)

Body weight is the ninth monitored parameter and contributes its four statistics within the window to $x_{\mathrm{vital}}$ as any other channel, which is where the 36 in the preceding section comes from. It also receives two features of its own, because its signal lives on a different timescale and those four statistics carry almost nothing. Heart rate and SpO_2_ change in seconds and are therefore summarised by within-window statistics; weight changes significantly only over days, and its absolute value carries much less information than its trend. Fluid retention preceding decompensation is the mechanism of interest: in patients receiving home telemonitoring, weight gradually increases over the month before a heart-failure hospitalisation and increases in the final week, and the chances of admission increase with the size of the gain [60].

Two trend features are therefore computed explicitly against the weight cache rather than over the five-minute window.

(2) $$\Delta w_{\tau }=\mathrm{clip}\;\left(\frac{w(t)-w(t-\tau )}{s_{w}},\;-1,\;1\right),\;\tau \in \left\{24\;h,\;72\;h\right\},$$

with scale $s_{w}=10$ kg. Both are signed, so the diuretic response appears as a negative excursion and is distinguishable from retention rather than collapsed into magnitude. When no prior weight is available, the deltas are set to zero and the missingness indicator for weight is increased, so that the model can distinguish a stable weight from an unobserved one.

This inclusion also makes the remote monitoring extension of Section 3.6 operative rather than nominal. A smart scale is the cheapest and most widely deployed device in a post-discharge heart failure kit, and without a weight channel in the feature vector, this device could not contribute anything to the risk score. However, its measurements are reliable.

### 4.3. Laboratory Features ($x_{\mathrm{lab}}$)

Laboratory values operate on a fundamentally different timescale from vital signs: while heart rate and SpO_2_ respond to physiological change within seconds, cardiac biomarkers, electrolytes and acid-base indices typically evolve over hours to days. The 12 laboratory parameters in $x_{\mathrm{lab}}$ were selected to provide complete coverage of the five physiological subsystems most predictive of cardiac deterioration [47]: myocardial injury and haemodynamic stress (troponin I, NT-proBNP), renal-cardiac interaction (creatinine), electrolyte balance (potassium, sodium, magnesium), acid-base and respiratory status (bicarbonate, pCO_2_, pH, lactate), and haematological status (hematocrit, WBC). Their LOINC codes and units are listed in Table 6. Serial trends matter more than single values for most of these: a rise in troponin indicates ongoing necrosis, natriuretic peptides are informative even as a single value, a BNP above 100 pg/mL identifying heart failure as the cause of acute dyspnoea with 83% accuracy [61]; and in severe sepsis and septic shock, lactate clearance below 10% over the first six hours of resuscitation is associated with higher mortality [62]. This motivates the serial delta features defined in this subsection.

#### 4.3.1. Temporal Dynamics and Look-Back Window Design

Laboratory values are held in the Faust RocksDB-backed cache with a 24-h rolling look-back, a horizon chosen to balance three constraints. Circulating cardiac biomarkers turn over on a timescale of hours, so older values carry diminishing relevance; conversely, electrolyte and CBC panels are ordered on 8 to 12 h schedules, so a shorter window would produce high missingness overnight and on weekends. The Acid-base parameters are the interesting case: ABG panels are typically drawn in response to a suspected deterioration event, so the absence of a recent ABG is itself evidence that the clinical team has not detected one. This informative missingness is explicitly captured by the indicator features of Section 4.4 rather than being imputed away.

#### 4.3.2. Physiological Interaction Features, and a Defect in One of Them

Two composite scores are carried as dedicated elements rather than removing raw measurements, giving $|x_{\mathrm{lab}}|=12+3+2=17$. One of them is sound; the other, as implemented and evaluated here, is not, and this section states both clearly.

Cardiorenal index (BCR). The sound one is the ratio of NT-proBNP to creatinine,

(3) $$\mathrm{BCR}=\frac{\left[\mathrm{NT}-\mathrm{proBNP}\right]}{\max([\mathrm{Creatinine}],\;0.1)},$$

carried as log(1+BCR) normalised to [0,1]. It rises with the release of natriuretic peptides and falls as renal function declines, separating cardiac decompensation with preserved renal function from cardiorenal syndrome in which both move together. Both inputs are measured directly and LOINC-mapped (Table 6).

The blood-urea-nitrogen-to-creatinine ratio, a conventional marker of pre-renal physiology, is not available in this feature vector. BUN is not among the twelve laboratory channels mapped, and it is not recoverable from creatinine and eGFR: the CKD-EPI equation computes eGFR from creatinine, age and sex and does not carry urea information. The implemented feature is the NT-proBNP ratio defined above, which carries a different clinical meaning and should not be read as a substitute.

The feature labelled SIG is degenerate and contributes nothing. The Stewart model defines the strong ion gap as a marker of unmeasured anion accumulation, and requires measured strong ions together with weak acid buffers. Chloride is not among the laboratory channels mapped, and the implemented feature is not the Stewart quantity, nor an anion gap, nor an approximation of either. What is computed is

(4) $${\mathrm{SIG}}_{\mathrm{impl}}=\mathrm{clip}\;\left(\frac{\left[{\mathrm{Na}}^{+}\right]+\left[K^{+}\right]-\left[{\mathrm{HCO}}_{3}^{-}\right]-12}{30},\;-1,\;2\right)/2,$$

which carries no chloride term and in which the constant 12 is not a chloride estimate.

More seriously, Equation (4) saturates. At normal values ($\left[{\mathrm{Na}}^{+}\right]$ 140, $\left[K^{+}\right]$ 4, $\left[{\mathrm{HCO}}_{3}^{-}\right]$ 24 mmol/L), the quantity within the clip is 3.6, well above the ceiling of 2, so the feature returns 1.0. Falling below that ceiling requires $\left[{\mathrm{Na}}^{+}\right]+\left[K^{+}\right]-\left[{\mathrm{HCO}}_{3}^{-}\right]<72$, which at a plausible sodium demands a bicarbonate above 72 mmol/L. Evaluated across the full physiological range that the pipeline admits, the feature is identically 1.0.

Three consequences follow, and we state them rather than quietly correcting the feature, because the reported results were produced with it in place. First, this column is one of three that does not vary in the evaluated matrix; Table 7 reconciles the count, which is 83 varying columns of 86 and 82 distinct ones. Second, a constant feature cannot influence any prediction, so the ablation and discrimination results reported in Section 6 and Section 9 are unaffected in substance: removing it would not change them. Third, any SHAP attribution reported against this position is meaningless, and it should be ignored wherever it appears.

Approximating chloride from an anion gap estimated via the Henderson-Hasselbalch relationship does not rescue the feature: substituting that approximation into the Stewart definition returns the estimated anion gap itself, so the construction is circular.

The feature that the available panel can support is instead the albumin-unadjusted anion gap, $\left[{\mathrm{Na}}^{+}\right]+\left[K^{+}\right]-\left[{\mathrm{Cl}}^{-}\right]-\left[{\mathrm{HCO}}_{3}^{-}\right]$, which requires the addition of chloride (LOINC 2075-0) to the extract. The implementation provides this behind a configuration flag, disabled by default so that the results reported here remain reproducible; enabling it changes the feature and therefore requires retraining. The constant-column check in the retraining pipeline, which previously logged a warning, now halts the run, so a feature in this condition cannot reach a trained model again without being overridden deliberately.

#### 4.3.3. Normalisation

Every feature is assigned to [0,1] by clipping a fixed physiological range and then linearly scaling within it, except the two signed weight-trend features of Section 4.2, which are clipped to [−1,1]:

(5) $${\tilde{x}}_{j}=\frac{\mathrm{clip}\left(x_{j},\;\ell_{j},\;u_{j}\right)-\ell_{j}}{u_{j}-\ell_{j}},$$

where $\left[\ell_{j},u_{j}\right]$ is a clinically chosen range for the parameter *j*, for example [0,20] mg/dL for creatinine and [100,180] mmol/L for sodium. The bounds are fixed in the implementation rather than estimated from the cohort.

No percentile is computed anywhere in the pipeline. The bounded transform is preferred to a robust scaler centred on the median and divided by the interquartile range for a reason the robust scaler does not share: both the sequence models and the alert threshold assume a bounded input domain, and a robust scaler leaves extreme laboratory values unbounded.

The trade-off this makes should be stated because it is the mechanism behind a defect reported in Section 4.3.2. Clipping is robust to outliers that characterise critical-care laboratory distributions, where troponin I spans several orders of magnitude and NT-proBNP reaches 70,000 pg/mL, and it guarantees the range gate that the training pipeline enforces. But it discards all information above $u_{j}$: a creatinine of 20 mg/dL and one of 30 mg/dL are the same input to the model, and any feature whose plausible values lie wholly outside $\left[\ell_{j},u_{j}\right]$ becomes a constant. Choosing $\left[\ell_{j},u_{j}\right]$ per feature is therefore a modelling decision with clinical consequences, and the implementation limits were set by physiological plausibility rather than derived from the cohort’s own distribution. Deriving them from training quantiles, for instance the first and 99th percentiles, is a straightforward improvement that we have not made.

#### 4.3.4. Missing Value Imputation

Missing laboratory values are a ubiquitous feature of real-world clinical datasets [30,63,64]. In the MIMIC-IV cardiac ICU cohort used for training, the missingness rates varied substantially between parameters: troponin I was available in 94% of the 5-min inference windows with at least one previous lab draw, while arterial pH was available in only 38% of the windows, reflecting its dependence on an arterial line and a clinical decision to obtain an ABG.

MedROAD V3 employs a two-stage imputation strategy:

Stage 1—Recency-weighted carry-forward. For a laboratory value missing at inference time *t*, the most recent available value $x_{j}\left(t^{\prime }\right)$ within a parameter-specific maximum look-back window $L_{j}$ is used, weighted by a linear decay function.

(6) $${\hat{x}}_{j}\left(t\right)=x_{j}\left(t^{\prime }\right)\cdot \left(1-\frac{t-t^{\prime }}{L_{j}}\right),\;t-t^{\prime }\leq L_{j}$$

The windows $L_{j}$ are set to reflect the physiological half-life and clinical ordering frequency of each parameter: L=48 h for troponin I and NT-proBNP, L=24 h for creatinine, electrolytes and haematological parameters, and L=8 h for acid-base parameters (bicarbonate, pCO_2_, pH, lactate), which change rapidly in de-compensating patients.

Stage 2—Midpoint imputation. When no value is available within $L_{j}$, the feature is set to the midpoint of its clipping range, which under Equation (5) maps to 0.5. The imputed value is the midpoint of a clinically chosen range rather than an estimated central tendency, and it maps to the centre of the unit interval rather than to its lower bound. The practical consequence is that a missing feature presents as an unremarkable mid-range value rather than as a null input, so the model cannot distinguish absence from an average measurement on that channel alone. The corresponding missingness indicator in $x_{\mathrm{miss}}$ then signals to the base learners, which are the models that see the feature vector, that this feature is imputed, allowing the LSTM and Transformer models—which receive the full 86-element vector—to weigh it accordingly.

Table 8 summarises the missingness rates and imputation parameters for each laboratory feature in the MIMIC-IV training cohort.

The high missingness rates for acid-base parameters (pCO_2_: 61%, pH: 62%, lactate: 55%) are consistent with the reported prevalence of arterial line placement in the MIMIC-IV ICU cohort and reflect the fact that ABG measurements are ordered in response to clinical concern–introducing information rather than missing-at-random (MAR) missingness. The stage-2 midpoint imputation for these parameters is therefore a conservative fallback; the missingness indicator in $x_{\mathrm{miss}}$ preserves this information for the downstream ensemble models.

### 4.4. Metadata Features ($x_{\mathrm{meta}}$)

Metadata features encode information about the data generation process rather than direct physiological measurements. In the clinical informatics literature, this class of features is increasingly recognised to carry independent predictive value beyond the physiological signals themselves, because systematic patterns in document timing, data completeness and intensity of care reflect clinical workflow states that strongly correlate with patient acuity [30,63,65]. MedROAD V3 includes $|x_{\mathrm{meta}}|=8$ metadata features, expanded from the original three, structured into four conceptual groups: data coverage, documentation timing, intensity of care, and patient temporal context. Table 9 provides a complete specification.

The eight metadata features are defined as simple normalised statistics over the inference window. The two coverage features $x_{\mathrm{vital}\_\mathrm{cov}}$ and $x_{\mathrm{lab}\_\mathrm{cov}}$ are the fraction of the 9 vital and 12 laboratory LOINC types, respectively, for which at least one observation is available, the late over a 24-h look-back. $x_{\mathrm{abg}\_\mathrm{present}}$ is set when pH, pCO_2_ and lactate were drawn within 4 h. The two timing features normalise the age of the most recent nursing note and of the most recent observation of vital-signs by the width of the window, which saturates at 1. The intensity of care is captured by $x_{\mathrm{med}\_\mathrm{burden}}$, the active medication count capped at 20 and divided by 20, and the binary $x_{\mathrm{iv}\_\mathrm{drip}}$. The temporal context of the patient is $x_{\mathrm{icu}\_\mathrm{los}}=\log\left(1+\min\left(\mathrm{LOS},30\right)\right)/\log\left(31\right)$, which compresses the long right tail of the ICU stay durations.

These features encode documentation behaviour rather than physiology and are included because documentation behaviour is itself informative: a clinical team that increases charting frequency and orders an ABG is responding to something the recorded values have not yet shown. Their interactions carry most of this signal. A growing documentation gap combined with a decrease in vital coverage indicates a unit under staffing pressure, during which deterioration is more likely to go unobserved. A recent ABG, together with an active infusion, marks active hemodynamic management. Low laboratory coverage late in an ICU stay suggests de-escalation toward discharge. Because these features are reported through the same SHAP pathway as physiological features (Section 5), a clinician can see directly when an alert is driven by data quality rather than by the patient.

#### Updated Feature Vector Dimensionality

With the expanded metadata sub-vector ($|x_{\mathrm{meta}}|=8$, up from 3), the full feature vector dimensionality becomes:

(7) $$D=|x_{\mathrm{vital}}\left|+\right|x_{\mathrm{lab}}\left|+\right|x_{\mathrm{weight}}\left|+\right|x_{\mathrm{miss}}\left|+\right|x_{\mathrm{time}}\left|+\right|x_{\mathrm{meta}}|=36+17+2+21+2+8=86$$

All inputs to the model, SHAP computations, and calibration procedures described in Section 5 and Section 6 are updated to D=86 accordingly. Table 10 states the complete schema and what each learner receives, so that the model can be reconstructed from the manuscript without reference to the source: the three base learners all consume the same 86 features, differing only in whether they see a single window or a sequence of them, while the meta-learner consumes three numbers, the calibrated base probabilities, and never sees the feature vector.

## 5. Ensemble Inference and SHAP Explainability

### 5.1. Ensemble Architecture

The feature vector $x\in R^{86}$ is evaluated in parallel by three base models. Only the LSTM is carried over from the published MedROAD V2 [10]; the gradient-boosting component is replaced by XGBoost with a calibration stage, the Transformer is new, and the V2’s isolation forest and autoencoder, which served anomaly detection and pattern recognition rather than supervised prediction, are not retained. All three are trained on the expanded feature set:

1. XGBoost classifier $f_{\mathrm{XGB}}$: trained in MIMIC-IV cardiac ICU admissions with binary outcome, deterioration in 12 h, defined as the first occurrence of initiation of vasopressor or inotrope (norepinephrine, epinephrine, vasopressin, dopamine, or dobutamine), invasive mechanical ventilation or death in the ICU. Events within six hours after admission to the ICU are suppressed, as patients arriving from the theatre are routinely ventilated and supported and would otherwise be labelled deteriorating upon arrival. Phenylephrine and milrinone are excluded from the definition of vasopressors: the former is ordered for the vast majority of this cohort for transient hypotension and the latter is commonly started electively in heart failure, so neither mark acute decompensation. Training used 5-fold cross-validation with early stopping on log-loss and grouping by patient throughout.
2. LSTM network $f_{\mathrm{LSTM}}$: processes the five-minute window in context as a sequential input $X\in R^{T\times 86}$, where *T* is the number of consecutive windows in the sequence. Architecture: 2-layer LSTM with hidden dimension 128, dropout 0.3, trained with AdamW and cosine learning rate schedule.
3. Transformer encoder $f_{\mathrm{TF}}$: processes the same sequence with 4 attention heads, $d_{\mathrm{model}}=64$, 2 encoder layers, and sinusoidal positional encodings.

The three outputs are combined by a logistic regression meta-learner trained on out-of-fold predictions:

(8) $$R=\sigma \;\left(w_{0}+w_{1}f_{\mathrm{XGB}}\left(x\right)+w_{2}f_{\mathrm{LSTM}}\left(X\right)+w_{3}f_{\mathrm{TF}}\left(X\right)\right)$$

where σ(·) denotes the sigmoid function and R∈[0,1] is the ensemble risk score. An alert is triggered when R≥τ. The Post-hoc calibration of each base model and the meta-learner is addressed in Section 6.

### 5.2. SHAP-Based Feature Importance

SHAP was introduced in the published MedROAD V2 [10], which applied TreeSHAP to its gradient-boosting component and DeepSHAP to its neural components, as a post-hoc global explainability tool. MedROAD V3 promotes it to a per-inference component whose output directly conditions the Claude API narrative (Section 7), making explainability an active part of clinical communication rather than a diagnostic aid available only to system developers. For every inference call with R≥τ, SHAP TreeExplainer is applied to $f_{\mathrm{XGB}}$ to compute Shapley values at the instance-level $\phi =(\phi_{1},\ldots ,\phi_{86})$ satisfying the following:

(9) $$f_{\mathrm{XGB}}\left(x\right)=\phi_{0}+\sum_{j=1}^{86}\phi_{j}$$

where $\phi_{0}$ is the base prediction (population mean) and each $\phi_{j}$ represents the marginal contribution of the feature *j* to the prediction for this instance. The top-5 features of $|\phi_{j}|$ are extracted and passed to the Claude API prompt, providing the attending physician with a physiologically grounded reason for the alert.

#### The Background Set and Attribution Stability

A Shapley value is defined relative to an expected-value baseline, and that baseline is estimated from a sample of training windows. The sample is therefore not an implementation detail: it partly determines which features a narrative names at the bedside, and it is reportable.

The background is 100 training windows drawn uniformly at random under a fixed seed. It is not stratified, and we withdraw the earlier description of it as “representative”, which implied a stratification that is not performed. With a window-level event rate of 4.4%, a uniform sample of 100 carries between four and five positive windows, so the baseline is dominated by non-event physiology and attributions are read against a typical rather than an average-risk patient. That is a defensible choice and arguably the right one for a bedside explanation, but it is a choice and condition for every attribution reported in this paper.

The attribute stability is now measured rather than assumed. Table 11 reports mean top-five overlap and rank correlation across ten background redraws at each of five sample sizes, with the coefficient of variation of the attributions broken out for the eight metadata features.

Three readings follow, and the second contradicts what we expected.

The configured size of 100 is where the curve flattens. The Top-five agreement rises from 0.776 at 25 to 0.869 at 100, gains 0.013 at 200, and falls back at 500. The rank correlation behaves the same way, peaking at 0.938. Therefore, The choice of 100 is supported by measurement rather than inherited, as previously was.

The metadata features are the most stable, not the least. We argued above that their near-constant values would make their small contributions the ones with a shifting baseline reorder most readily. The opposite holds: their coefficient of variation is 0.049 at the configured size versus 0.229 across all features, a factor of 4.7 and the ratio is between 3.1 and 7.6 at every size tested. Near-constant inputs evidently produce consistently small and consistently signed attributions, where physiological channels with wide value ranges vary more between baselines. The question was reasonable and wrong, and we report it as such because the reasoning behind it is the kind of thing a reader would otherwise repeat.

What remains unsatisfactory is that the top-five agree at the configured size is 0.869, which is 4.65 features in common out of five. Roughly one feature in the named set changes between redraws. The narrative names two and the stability of top two specifically is not what this measurement reports; a top-five overlap can hide disagreement at the head of the ranking. Given that the leading attribution is a metadata feature in 42% of windows (Section 7.3), the quantity that matters for the bedside explanation is the stability of the first two positions, and measuring it is the next step rather than a completed one. The variation in CV across sizes is also non-monotonic, which ten redraws is too few to resolve.

SHAP TreeExplainer uses this background set, reducing the per-inference overhead to 2.55 ms at the median (Section 9.4) while maintaining the Shapley consistency guarantee [35].

### 5.3. Alert Threshold Calibration

The published MedROAD V2 [10] selects an operating point on the uncalibrated ensemble score and reports a positive predictive value of 0.42 there, compared to 0.22 for NEWS2 in the same cohort. What V2 does not report is probability calibration, so its operating point is a rank cut rather than a probability, and the value of the score itself carries no quantitative interpretation. MedROAD V3 replaces this with a calibrated maximum F1 threshold. The threshold τ is set at the maximum operating point $F_{1}$ in patients who were left out, so that positive predictive value enters the choice of operating point; low-specificity alerts are those clinicians learn to override, and reducing inappropriate alerts is the remedy Nanji et al. propose for alert fatigue [44].

The value of τ does not transfer across cohorts, and this is worth stating plainly because it is easy to assume otherwise. Applying the configured constant τ=0.72 to the present cohort yields $F_{1}=0.078$: the threshold sits so far in the upper tail of the recalibrated score distribution that almost no window reaches it. The failure is neither discrimination nor calibration, both of which are intact on that split; it is the operating point alone. The reason is that an operating point is a property of a score distribution and an event rate, and both differ between cohorts.

This is the concrete form of the distributional shift argued for in Section 6.3. A site inheriting a threshold without re-deriving it from its own outcomes will not see degraded ranking or miscalibrated probabilities; it will simply see an alerting system that rarely alerts, which is the failure mode least likely to be noticed. Every deployment must re-derive τ from local data, and the reported value should be read as the procedure’s output on one cohort rather than as a constant of the system. The 0.72 used in this demonstration was never fitted to any cohort (Section 7.7); it is used here only to show what inheriting a threshold does.

## 6. Model Calibration

Discrimination is the ability of a model to classify high-risk patients above low-risk patients, measured by AUROC, which is a necessary but insufficient criterion for clinical deployment. However, A model with excellent discrimination can assign systematically biased probability estimates: a predicted risk of 0.72 may correspond to a true event rate of 0.30 or 0.90, depending on the population and training protocol. In a clinical decision support context, calibration is the alignment between predicted probabilities and observed event rates, which is arguably more consequential than discrimination, because it determines whether the risk score *R* presented to the physician has a meaningful quantitative interpretation [66,67]. This section formalises the calibration problem in the MedROAD V3 context, defines the metrics used to measure it, describes the post-hoc calibration methodology applied to each base model and the meta-learner, and addresses the particular challenge of calibration drift under distributional shift when the system is deployed at the SERVIER Virtual Cardiac Centre.

### 6.1. Calibration Metrics

Let $R_{i}\in \left[0,1\right]$ be the ensemble risk score and $Y_{i}\in \left\{0,1\right\}$ be the binary outcome of deterioration of the patient-window *i*. A perfectly calibrated model satisfies:

(10) $$P\left(Y_{i}=1|R_{i}=r\right)=r\;\forall \;r\in \left[0,1\right].$$

In practice, calibration is assessed over discrete probability bins $\left[b_{k-1},b_{k}\right)$, k=1,…,K. MedROAD V3 uses K=10 equal-width bins of width 0.1. For each bin, the mean predicted probability ${\bar{R}}_{k}$ and the observed event fraction ${\bar{Y}}_{k}$ are compared.

Partitioning the *N* inference windows into M=10 equal-width confidence bins $B_{1},\ldots ,B_{M}$, the expected calibration error is the sample-weighted mean gap between accuracy and confidence,

(11) $$\mathrm{ECE}=\sum_{m=1}^{M}\frac{|B_{m}|}{N}\left|\mathrm{acc}\left(B_{m}\right)-\mathrm{conf}\left(B_{m}\right)\right|,$$

and the maximum calibration error is the worst such gap, $\mathrm{MCE}=\max_{m}\left|\mathrm{acc}\left(B_{m}\right)-\mathrm{conf}\left(B_{m}\right)\right|$. ECE summarises average miscalibration; MCE exposes the single worst-behaved confidence region, which matters wherever an operating point sits in a sparsely populated region because a bin holding few windows can hide a large error. The MCE is computed by the evaluation code but is not tabulated here; the reliability diagram below carries the same information for the regions that matter.

The Brier score $\frac{1}{N}\sum_{i}{\left({\hat{p}}_{i}-y_{i}\right)}^{2}$ is also reported throughout, a proper scoring rule combining calibration and discrimination that cannot be improved by sacrificing one for the other. Its decomposition into reliability, resolution, and uncertainty terms, and the Hosmer-Lemeshow goodness-of-fit test over deciles of predicted risk, are both informative here and neither is reported in this paper; we note them as the obvious additions rather than imply that they were computed.

### 6.2. Calibration of Individual Base Models

Gradient boosting models such as XGBoost are known to produce poorly calibrated probability estimates, particularly in low- and high-probability tails [68]. The sigmoid output of the XGBoost model is applied to the raw leaf-sum prediction, which tends to produce overconfident estimates (probabilities clustered near 0 and 1) when the model is trained with default regularisation.

LSTM and Transformer models produce probabilities through a sigmoid output layer and are susceptible to overconfidence arising from overparameterization relative to the size of the training dataset. This is a well-documented phenomenon in deep learning [66]. Temperature scaling is applied post-hoc to both neural models.

Each component is calibrated by the method chosen for its output geometry, all fitted on a held-out validation split never used for training. XGBoost receives Platt scaling, a two-parameter sigmoid ${\hat{p}}_{\mathrm{cal}}=\sigma \left(a\cdot \mathrm{logit}\left(\hat{p}\right)+b\right)$, which suits gradient-boosted trees whose uncalibrated scores are already approximately sigmoidal in log-odds [68]. The LSTM and the transformer receive temperature scaling, a single-parameter softening ${\hat{p}}_{\mathrm{cal}}=\sigma \left(\mathrm{logit}\left(\hat{p}\right)/T\right)$ fitted by minimising negative log-likelihood [66]; one parameter is appropriate because neural overconfidence is a systematic effect rather than a distribution-specific distortion. The meta-learner receives isotonic regression, a non-parametric monotone fit [69] that is the strongest of the three but the most data-hungry, which is why it is applied only at the ensemble stage where the full validation set is available.

All three transformations are monotone, so none alters AUROC. Calibration changes what the score means, not how well it ranks.

That distinction has a limit, and the two claims should not be run together. Calibration concerns the meaning of a probability: a calibrated 0.15 denotes a 15% chance of the event, and where calibration holds at a second site, it denotes the same thing there. It says nothing about the transportability of a decision threshold. Where τ should fall depends on the local event rate, on the shape of the local score distribution, and on the local ratio of the cost of a missed event to the cost of a false alert, and all three can move between sites while the calibration is perfectly stable. Section 5.3 demonstrates exactly that: a threshold carried between cohorts fails outright while discrimination and calibration both survive. Calibration therefore makes a probability comparable between sites and makes a threshold derivable from local data; it does not make a fixed threshold portable, and every deployment must re-derive its own by the procedure of Section 7.7.

Table 5 reports discrimination and calibration for each base model, every leave-one-out ensemble, the full ensemble, and a logistic regression on the same split as a reference scorer. It is calculated on the reported cohort of Section 9.3: full cardiac units with a blank six-hour post-admission window, 25,214 stays of which 5907 (23.4%) carry an event, producing 1.75 million five-minute windows. The ablation refits every model on its own grouped split and evaluates on 435,307 held-out windows, 19,285 of them positive, a window-level event rate of 4.4%. Section 9.3 sets out that label and its partitions.

The degradation experiment of Section 3.6 uses a logistic scorer rather than the ensemble, deliberately, so that the measured loss reflects the restriction of features rather than differences in the capacity of the model, and its reference figure is therefore not comparable with the figures of the ensemble here.

Four observations follow. First, the complete ensemble attains the highest AUROC at 0.816, and all reduced variants fall below it. The margins are narrow: dropping the Transformer costs 0.008 AUROC, and dropping either the LSTM or XGBoost costs 0.002.

Second, a regularised logistic regression on the identical grouped split reaches 0.758, compared to 0.816 for the ensemble, a difference of 0.058 at p<0.001. This answers a question the comparison with NEWS and MEWS does not answer: beating a severity score designed in the 1990s establishes less than beating a linear model given the same 86 features and the same patients. The ensemble clears that bar. Its calibration is no better than the linear model’s, however, at ECE 0.001 against 0.002, so the gain is in discrimination alone.

Third, individual learners are closely matched in discrimination, 0.805 to 0.810, but differ by two orders of magnitude in calibration. The LSTM and Transformer carry ECE of 0.334 and 0.372 after temperature scaling, while any ensemble containing at least two members reaches 0.001 to 0.002.

That gap is not a tuning failure, but a structural one, and its mechanism settles whether temperature scaling is the method best suited to these outputs. Under this training regime, it is not. Temperature scaling fits a single multiplicative scalar. Training under a positive class weight large enough to counter a 4.4% event rate shifts the logits additively, and no scalar divisor removes an additive shift. The ensemble’s calibration therefore owes nothing to the deep models’ own calibration stage, and the three-stage pipeline this paper describes is in practice a one-stage pipeline with two inert stages in front of it. The implementation now offers an affine alternative, fitting a scale and an intercept, which is Platt scaling on the logits and removes the shift. Every figure in this paper is from the temperature-only path, which we verified by reading the fitted intercept out of the saved checkpoints: it is zero in both deep models.

Therefore, two configurations must be distinguished. The evaluated pipeline is Platt, temperature and isotonic, which produced every number reported here; in it the deep stage is inert and the three stages are in practice one, the averaging and the isotonic step in the ensemble doing the work between them. The recommended pipeline replaces the temperature with the affine fit in the deep stage. We report the evaluated one because switching would move every table without moving the number a clinician sees, and would replace a measured result with an unmeasured one. A deployment that uses the deep models individually rather than only through the ensemble must enable the affine fit, as must any deployment that drops the isotonic stage; one using the ensemble as evaluated here may leave it alone.

Where the abstract and Section 1 describe a complete calibration pipeline, they describe what is implemented; what is demonstrated is the ECE of the ensemble.

**Figure 2:**
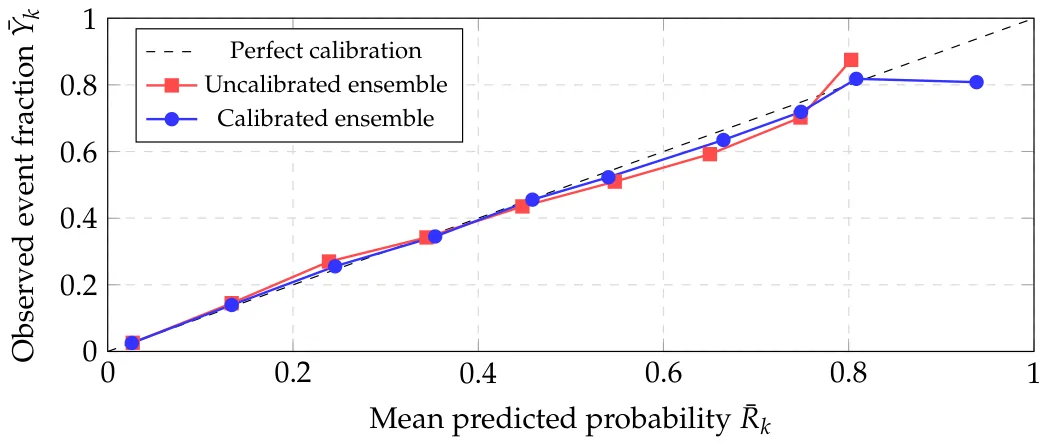
Reliability diagram for the ensemble on held-out patients (n=435,307 windows, 19,285 positive). Bins containing no observations are omitted. Points above the diagonal indicate underestimated risk, below it overestimated.

Fourth, and contrary to what the ECE figures alone suggest, the reliability diagram (Figure 2) shows that most of that calibration comes from averaging rather than from the isotonic step.

Both curves closely track the diagonal across the populated range and are difficult to distinguish. The meta-learner’s output is already near-calibrated before isotonic regression is applied, because averaging three models with differently-shaped probability surfaces cancels much of the individual distortion. The isotonic step then refines what remains, improving the ECE from roughly 0.009 to 0.001, a real but modest absolute gain. The reported ECE of 0.001 should therefore be read as a property of the ensemble as a whole rather than as evidence that post-hoc calibration performs the transformation; the transformation that matters is the average.

Two other features of the diagram deserve to be noted and both concern its top end. The two curves do not reach the same place: the uncalibrated ensemble stops at a highest bin of predicted 0.803 against observed 0.875, underestimating risk there, while isotonic regression stretches the top of the range to a bin at predicted 0.938 against observed 0.808, which overestimates it. Between predicted 0.55 and 0.75 both curves sit slightly below the diagonal, and the isotonic step narrows but does not close the gap. Those top bins hold very few windows, so neither tail figure should be read as a calibration result.

This matters little for the operating point. The derived threshold of Section 7.7 is 0.153, and 0.023 at 90% sensitivity, both of which sit in the densely populated lower half of the distribution rather than in the sparse tail where the two curves diverge. A threshold chosen in that tail, such as the configured constant of 0.72 discussed in Section 7.7, would sit in exactly the region described in this paragraph.

#### What Table 5 Does and Does Not Establish

Returning to the first of these four observations: the case for retaining each member rests on the leave-one-out margins reproducing, not on the *p*-values beside them. Across the four independent retrainings of Table 12 each margin is positive every time and the ordering is identical, which is evidence that does not depend on an independence assumption. The DeLong *p*-values beside the table are reported for continuity with the literature and are not load-bearing. The clustered *p*-values given in the following are, and they agree.

Two qualifications apply to every row of Table 5, and together they bound what it establishes far more tightly than the *p*-values suggest. We state them here rather than in a limitation, because a reader who takes those *p*-values at face value will overread the table.

The window is not the sampling unit. Every interval in Table 5, and DeLong’s test beside it treats a five-minute window as an independent observation. The 435,307 holdout windows come from 5049 patients, so a patient contributes scores of windows that share physiology, charting practice and a single outcome. The effective sample size is closer to the patient count than the window count, and the standard error is understated by a factor that grows with the correlation within the patient. The effect of design for a clustered estimate is 1+(m−1)ρ with *m* the size of the cluster and here m=86 windows per patient. The measured widening is the cleanest way to read it: an interval 3.8 times wider is a variance 14 times larger, which at m=86 implies a correlation within the patient of approximately 0.16. That is a high but unsurprising value for an outcome which is a property of the stay rather than of the window.

The factor is now measured rather than argued, and the clustered inference has been run on every row. Table 13 gives it. The estimates of points are unchanged, as resampling patients does not move them; the intervals are increased by between 3.6 and 3.9 times, so every interval in Table 5 should be read as close to four times wider than printed. At that width the full ensemble and all three reduced variants overlap one another comfortably, which is what the window-level intervals concealed.

Clustered *p*-values are the more informative column, and do not overturn ablation. Every reduced variant still differs from the full ensemble, but one comparison weakens far more than the rest: dropping the LSTM moves from p<0.001 under DeLong to p=0.016 under patient resampling, an order of magnitude, while the others remain at the resolution floor of the bootstrap. The margins LSTM and XGBoost are equal in this table at 0.002, so it is not the smaller margin that clustering punishes, but the one whose advantage is less evenly distributed between patients. That floor is worth naming rather than letting a reader read 0.002 as a measurement: with 500 patient resamples the smallest attainable two-sided *p*-value is 1/500, and a row showing 0.002 means only that no resample reversed the sign of the difference. Resolving those rows further would require more resamples, which we have not run because nothing in the paper explains whether they are 0.002 or smaller.

The conclusion is therefore narrower than the original *p*-values suggested and survives in substance. Treating the patient as the sampling unit costs roughly a factor of four in every interval and an order of magnitude in the LSTM’s value *p* and leaves the ordering of the ablation intact.

The training run is held fixed. DeLong’s test is paired on the same windows and on the same fitted models, so it resolves whether these two fits differ in this population. It is silent on whether the architectures differ, and Section 7.8 measures a retraining spread of up to 0.008 AUROC in deep learners, which equals or exceeds every leave-one-out margin in this table.

A *p*-value below 0.001 against a margin of 0.002 is therefore a statement about two particular fits. The independence problem is now repaired rather than flagged, and the repaired inference reaches the same ordering at wider intervals and a weaker *p*-value for the LSTM. What remains unrepaired is the second qualification: every *p*-value condition in both tables, clustered or not, in one training run, so none of them is evidence of the architecture-level. For that the paper relies on the four retrainings of Table 12.

However, the leave-one-out margin is the right quantity to look at, and it is better behaved than the level. Within a run, the full ensemble and each reduced variant share their base-model fits, so the margin is paired, and the level’s variance does not enter it. Table 12 repeats the comparison under distribution shift in four independent retrainings, which is the test that this question needs, since a component that looks marginal in distribution need not be marginal out of it. Dropping the LSTM costs 0.003, 0.003, 0.005, and 0.001 AUROC across the four runs, positive in each but spanning a factor of five. That is modest evidence rather than proof, four runs being a small number and the spread among them wide, but it is consistent with the in-distribution result and it is not an artefact of a single fit. The authentic second site remains the experiment that would decide the question.

### 6.3. Distributional Shift and Site-Specific Recalibration

A well-calibrated model on MIMIC-IV can become severely miscalibrated when deployed at a different clinical site, because of distributional differences in patient demographics, comorbidity burden, care protocols, and documentation practices between institutions. This is one of the failure modes most frequently identified in clinical ML systems deployed [54,63,70].

Three mechanisms produce miscalibration under distributional shift in the MedROAD V3 context:

Covariate shift. The marginal distribution P(x) differs between the MIMIC-IV training site (Beth Israel Deaconess Medical Center, Boston) and the SERVIER deployment site. For example, SERVIER patients may have a higher baseline NT-proBNP due to a different referral mix (tertiary cardiac centre vs. general academic hospital), which would shift the predicted *R* upward without a corresponding increase in the true rate of events.

Label shift. The marginal event rate P(Y=1) may differ between sites. MIMIC-IV has a cardiac ICU deterioration event rate of approximately 4.4% per 5-min window; the SERVIER site event rate is unknown a priori and will be estimated from the first 500 patient-days of the prospective deployment data.

Concept drift. The conditional distribution P(Y∣x) can differ, for example, if the SERVIER site has more aggressive early intervention protocols that reduce deterioration events conditional on the same physiological state, the model will systematically overestimate risk.

#### Detection and Correction of Calibration Drift

The drift is monitored by computing the ECE over a rolling window of recent inferences for which outcomes have become available and comparing it against the baseline set for the validation. A sustained excursion beyond a preset tolerance triggers recalibration. Because all three calibration maps are monotone and cheap to refit, correction does not require retraining any base model: the isotonic fit at the ensemble stage is re-estimated on the accumulated local outcomes, leaving the learnt representations untouched. This is the lighter alternative to the federated retraining referred to in Section 2, and addresses the specific failure that matters operationally, that is, τ ceases to mean what it meant at the development site.

### 6.4. Threshold Calibration and the Sensitivity-Specificity Trade-Off

The choice of alert threshold τ directly determines the clinical operating point of MedROAD V3. Unlike threshold selection in standard ML evaluation, clinical threshold calibration must account for the asymmetric cost structure of false positives (alert fatigue, unnecessary interventions, staff desensitisation) and false negatives (missed deterioration events, delayed treatment). Let $c_{\mathrm{FP}}$ and $c_{\mathrm{FN}}$ denote the clinical costs of a false positive and false negative alert, respectively. The cost-optimal threshold minimises the expected cost per inference window:

(12) $$\tau *=\underset{\tau }{\arg\min}\;c_{\mathrm{FP}}\cdot \mathrm{FPR}\left(\tau \right)\cdot \left(1-\bar{Y}\right)+c_{\mathrm{FN}}\cdot \mathrm{FNR}\left(\tau \right)\cdot \bar{Y}$$

where FPR(τ) and FNR(τ) are the false positive and false negative rates at threshold τ, and $\bar{Y}$ is the site-specific event rate. For the reported cohort, with $\bar{Y}=0.044$, a cost ratio $c_{\mathrm{FN}}/c_{\mathrm{FP}}=5$ (reflecting the greater harm of a missed deterioration event) produces, for a calibrated score, $\tau *=c_{\mathrm{FP}}/\left(c_{\mathrm{FP}}+c_{\mathrm{FN}}\right)\approx 0.17$, which lies in the upper decile of the scoring distribution and close to the $F_{1}$-maximising 0.153 of Section 7.7.

Four assumptions are embedded in that expression and are worth stating, because each limits how far the resulting threshold travels. First, $\bar{Y}$ is a window-level event rate under labelling, not a patient-level incidence. The two differ by roughly a factor of five in the reported cohort of Section 9.3, where 4.4% of the windows and 23.4% of stay carrying an event. A site substituting its own incidence at the patient-level into this expression will obtain a threshold that is wrong in a direction it cannot see. Second, the expression treats every window as an independent decision, which the episode-level policy makes false in deployment: once consolidation is applied, the cost of a false positive is incurred per episode rather than per window, and τ* should be properly derived against the episode-level operating characteristic. We have not done so. Third, costs are assumed constant across patients and throughout the stay, whereas the harm of a missed event plausibly varies with how much physiological reserve a patient retains. Fourth, the ratio of 5 is illustrative. We are unaware of an empirical estimate of $c_{\mathrm{FN}}/c_{\mathrm{FP}}$ for cardiac ICU deterioration alerts, and the value should be elicited locally rather than inherited; the figure is used here only to show the form of the trade-off rather than to fix a value: τ* depends on the local event rate and on the shape of the local score distribution, and Section 5.3 shows empirically that a threshold carried over from another cohort performs poorly even when discrimination and calibration are both sound.

### 6.5. Class Imbalance and Its Effect on Calibration

The cohort exhibits a 4.4% event rate, roughly twenty-two non-event windows per event window. Trained naively, cross-entropy under this imbalance biases models toward low predicted probabilities, compounding the overconfidence described above. Three measures counteract it. XGBoost is trained with scale_pos_weight set to $\left(1-\bar{Y}\right)/\bar{Y}\approx 22$ [24], and the LSTM and Transformer apply the equivalent per-sample weighting to their binary cross-entropy. All cross-validation folds are stratified so that the event rate is preserved in every split, without which calibration estimates on small folds become unstable. Critically, resampling is not used: oversampling the minority class would distort the very base rate that calibration is intended to recover, leaving the calibrated score systematically overstated relative to true prevalence.

## 7. Clinical Narrative Generation via Claude API

### 7.1. Motivation and Scope

A risk score and its SHAP attributions summarise the deterioration signal quantitatively, but they are not a clinical communication. A score of 0.81 is not actionable until it is translated into the attending physician’s vocabulary: What abnormality is driving the alert, what it plausibly means given the medications and diagnoses of this patient, and what action is indicated. Contextualised multi-source summarisation of this kind is precisely where language models perform well [40,42].

MedROAD V3 therefore calls the Claude API conditionally, when R≥τ, to produce a structured three-sentence narrative accompanying the alert. The narrative is neither a diagnosis nor an order. It is an AI-generated decision support summary that requires physician review, consistent with the human-in-the-loop design discussed under the Health Canada SaMD framework [55] in Section 10, whose device class remains to be determined, and with published governance principles for language models in acute care [71].

### 7.2. Prompt Architecture

The Claude API call uses a two-component prompt structure: a system prompt that defines the model’s role, output constraints, and safety boundaries, and a user prompt that provides the patient-specific clinical context assembled from the current inference window. This separation follows the recommended practice for the deployment of LLM in context-constrained clinical settings [41,72,73] and ensures that the role specification and output format are consistent in all invocations regardless of the specific content of the patient.

#### 7.2.1. System Prompt

The system prompt is fixed at the time of deployment and the design requires six constraints. The model acts as a decision support assistant rather than a diagnostic physician, never stating a diagnosis. The output must be exactly three sentences: the current clinical picture, the main drivers identified by SHAP, and a concrete recommended action. The register is cardiologist-level, allowing standard critical-care abbreviations, since the output is directed at specialists and must be compact. If the active medication list contains a known interaction, such as amiodarone with warfarin or a QT-prolonging agent with hypokalaemia, a fourth sentence with an interaction alert is appended. Every response must contain the verbatim provenance string “AI-generated decision support summary, physician review required”, placed so that it survives copy-paste into handover notes. Finally, definitive phrasing is prohibited: the model is instructed to treat its output as a differential hypothesis, producing “findings consistent with early cardiogenic shock” rather than “the patient is in cardiogenic shock”.

The Listing 1 is what the evaluated implementation actually sends, and is shorter than that specification.
**Listing 1.** System prompt as deployed, verbatim.You are a clinical decision support system embedded in an ICU electronic health record. Your role is to generate concise, accurate, and~actionable clinical alerts. Always write exactly three sentences:  1. State the risk score and its clinical significance.  2. Name the two most important physiological drivers from the SHAP analysis.  3. Recommend the single most appropriate immediate clinical action. Use plain clinical language. Do not speculate beyond the provided data. Do not mention AI, machine learning, or~algorithms in the output.

Of the six constraints, the deployed prompt carries the three-sentence structure and the prohibition on self-reference, and states the decision-support role without explicitly forbidding a diagnosis. Three are absent. There is no instruction to hedge, so the prohibition on definitive phrasing is enforced neither at the prompt nor at the output. The deployed prompt asks for plain clinical language rather than the specified cardiologist-level register, which is a different instruction rather than a weaker one. And the fourth interaction sentence cannot be produced at all: no medication list reaches the prompt in this implementation, and the output contract validates for exactly three sentences, so a four-sentence response would be diverted to the fallback. The specified four-sentence form is therefore incompatible with the built validator, and reconciling them is a prerequisite for the medication-interaction feature.

The provenance string is, likewise, not produced by the model. It is attached downstream, by the write-back layer, which states in the SOAP note’s plan field that the content is machine-generated and requires physician review (Section 8.1). The effect is the same in the chart and the mechanism is more reliable, since it does not depend on the compliance of the model, but the manuscript should not describe it as a constraint.

#### 7.2.2. User Prompt Construction

The user prompt is assembled at inference time from five structured sources: the mean and rate of change of each of the nine vital signs over the current window; the five highest-magnitude SHAP attributions from the XGBoost explainer, given as feature name, value and Shapley contribution; the most recent laboratory values within the 24-hour look-back; the ensemble risk score alongside its three component scores and the threshold; and the active medication list with dose, route, and frequency, which is the minimum needed for interaction assessment. Every field is numeric or drawn from a controlled vocabulary. The free-text EHR content is not included, which prevents the injection pathway discussed in Section 7.13. The Listing 2 gives the template with variable fields in {curly braces}.
**Listing 2.** MedROAD V3 user prompt template for Claude API narrative generation.Case reference: {pseudonymised_id} ICU LOS: {icu_los_hours:.1f} h  |  Encounter: {encounter_id} Risk score: {R:.3f}  (XGB={xgb:.3f}, LSTM={lstm:.3f}, TF={tf:.3f}) Alert threshold: {tau:.2f} --- VITAL SIGNS (5-min window) --- {vital_table} --- TOP-5 SHAP FEATURES --- {shap_table} --- LABORATORY VALUES (most recent within 24h) --- {lab_table} --- ACTIVE MEDICATIONS --- {medication_list} Generate a structured clinical decision support summary. Three sentences required (four if drug interaction detected). Format: S1=clinical picture | S2=SHAP drivers | S3=action | S4=DDI (if any). Append: “AI-generated decision support summary -- physician review required.”

Two notes on this listing. The identification of patients is pseudonymised prior to transmission, as a salted digest, for the reasons given in Section 7.10. And the listing specifies the full prompt the design requires, whereas the implementation evaluated here populates a subset of it, the risk score and the SHAP attributions, and does not yet assemble the vital, laboratory, and medication tables. The narrative evaluation of Section 7.3 was therefore run against that subset rather than against the full specified prompt, and its grounding figures bound what the gates do on the fields actually supplied, not on the contract as designed. The Listing 3 in Section 7.14 shows a populated instance of the subset that is emitted, together with the model’s response to it, so the two can be compared directly.

### 7.3. Measured Narrative Output and What the Model Attends to

One hundred and eighty windows of patients who did not respond in the reported cohort were sampled above the threshold that gives 90% sensitivity (τ=0.023), narrated through the deployed prompt and scored against both runtime gates. Both gates accepted 173 and rejected 7, a rejection rate of 3.9%.

All seven rejections were false. Four were rejected by the contract for containing a demographic descriptor, the descriptor being “white” inside the “white blood cell count”—the white cell count is one of the twelve mapped laboratory channels, so the filter suppressed narratives to correctly name a feature the model uses. The other three scored 0.67 on grounding for the unmatched value 12, which is the prediction horizon the narrative states as “within the next 12 h”. The horizon is a property of the score and was not in the set of values the grounding check treats as supplied.

Both are fixed and both belong in the record because they are the third and fourth false rejections this filter has produced; the first two were the alert threshold and the pseudonymized case reference. Every rejection the grounding and contract checks have ever made on real output has been a false one. We therefore report no demonstrated detection rate in either direction and we regard measuring the false-rejection rate against annotated output as a prerequisite for presenting either gate as a safety mechanism.

The informative measurement is what the narratives were asked to describe. The prompt names the five largest SHAP attributions, and over these 180 windows the single largest is a metadata feature in 42% of cases and a missingness indicator in another 23%. The most frequent strongest driver is the time since admission to the ICU, which leads in 38% of the windows, followed by the absence of a lactate measurement at 22%. In 21% of the windows, neither of the two leading drivers is physiological at all, and in 18% the two are a laboratory channel together with its own missingness flag, a pair that cannot be narrated coherently. An example generated reads: “a reduced lactate value, which lowers the risk estimate, and a pattern of missing lactate measurements, which raises concerns.”

This is a finding about the model, not about the language layer. A deterioration score whose leading attribution is usually how long the patient has been in the unit, or whether a clinician chose to send a lactate, is in a substantial part reading care process rather than physiology. Ordering a lactate is an act of clinical concern, so its presence or absence carries information about what the team already believed, which is a path by which outcome information reaches the features without passing through the patient’s measurements. We did not design the metadata block with that in mind, and Section 7.6 sizes the channel directly: the twenty-nine care-process columns alone reach AUROC 0.767. It supports the claim that SHAP output constitutes a clinical explanation: an accurate attribution of the model can still be useless or misleading at the bedside.

What reaches the prompt, exactly. Because the attribution ranking is so often not physiological, the rule connecting the two matters, and we state it rather than leave it to be inferred. In the evaluated implementation there is no rule. The five attributions of the highest absolute magnitude were formatted and inserted verbatim, in rank order, under the header “Top physiological drivers”, and the system prompt asked for the two most important physiological drivers. No filtering, substitution, or reordering occurred at any point between the SHAP ranking and the prompt, so a metadata or missingness feature reached the model described as physiological whenever it ranked in the top five, which is most of the time in this cohort. Two defects in the formatter compounded this. It stripped the meta_ and miss_ prefixes, so meta_icu_los arrived as “icu los” and the model had no way to tell that the feature was not a measurement of the patient; and it rendered any delta_ feature as “rising” whatever the sign of the change, so a falling troponin was labelled a rising one, which is a factual error the lexical grounding check cannot detect because it compares numbers rather than words. The distributed implementation now tags each driver as a physiological or care process, keeps the prefixes on the label, words the direction so it cannot be read as the direction of the measurement, and instructs the model to name a care process driver as such.

The driver-selection statistic must be read against that. The narrative names the strongest driver in 45% of the generated cases and both two top two in 33%, and these figures cannot be attributed to the language model, because the model was asked for physiological drivers from a list that frequently contained none and was given no means of revealing which was which. Declining to call time since admission to a physiological driver is arguably the better behaviour. We do not present the remainder as failures, and we also do not present 45% as a measure of faithfulness. Separating a model that attends to the wrong thing from an interface that mislabelled what it was attending to requires rerunning the evaluation against the corrected prompt, which we have not done.

### 7.4. Informative Feature Count

The vector has 86 columns. Three of them do not vary in the data behind any result in this paper, and two more carry the same number as each other, so the effective input space is 83 varying columns and 82 distinct ones. Table 7 is the reconciliation, given because the figure has been stated inconsistently and because every SHAP attribution is read against it.

The four fail in different ways, and only one of them is harmless. The strong ion gap is constant for the reason set out in Section 4.3.2; a corrected implementation using chloride exists in the distributed code behind a configuration flag that was off for every run reported here, so the evaluated models carry the degenerate version. meta_med_burden and meta_iv_drip are constant because the training pipeline passes an empty medication list where the live path passes the real one, which is a train-serve skew rather than two wasted columns, and Section 7.5 states the deployment rule it forces. A constant input cannot influence a prediction, so no discrimination figure in this paper is affected by any of the three.

meta_vital_recency is not constant. The value of meta_doc_lag is assigned in the feature builder, the two having been intended as separate quantities and implemented as one, so the vector carries a perfectly collinear pair. This changes no prediction: a gradient-boosted tree splits on whichever it meets first, and the linear meta-learner never sees the feature vector at all. It does affect interpretation, because Shapley values divide credit between perfectly correlated inputs in a way that depends on the estimator rather than on the data, so an attribution reported at either index is half of a quantity belonging to both, and reading either position in isolation is unsound.

Five more columns are varying but fragile. The serial biomarker deltas depend on the ETL that has previous laboratory values, and are identically zero if it does not. Two body-weight statistics vary only over the full matrix: a validation gate reading first 200,000 rows alone reports them constant, which is a sampling artefact rather than a property of the feature.

### 7.5. The Medication Features and the Deployment Rule They Force

meta_med_burden and meta_iv_drip are built from a FHIR MedicationRequest list. The training ETL passes an empty one because the extract does not have prescription records, so both are identically zero in all 1.75 million training windows. The path of live inference passes the real list and would provide varying values. The two paths therefore disagree, and the disagreement runs in the dangerous direction.

A model cannot learn what a feature means from a column that does not vary. A gradient-boosted tree never places a split on it, so in inference the value is read and discarded. The deep models are worse placed: the input weights exist but were updated only by gradients computed at a single input value, so they are unidentified rather than zero, and presenting a non-zero value at inference drives the network with a quantity it has no fitted response to. The effect is unbounded in principle and unmeasurable here, since measuring it would require the varying values the extract does not contain.

The rule that this forces is narrower than saying that a deployment should populate the two or remove them, which is advice that can be followed in the wrong order. A deployed model must hold both features at the value they took during its own training, which is zero, regardless of what the live feed supplies, until a model has been refitted on an extract that genuinely carries prescription records. Populating them in the present models is not a partial improvement; it is the one action that introduces the skew. The implementation now enforces this rather than documenting it: the feature builder pins both columns and logs a warning when the live feed offers values, and the pin is released only by a configuration flag whose meaning is that the loaded model was trained with real medication data. Removing the columns outright is equally sound and requires a refit, which is why pinning is the default.

### 7.6. Care Process as a Predictor, and What It May Encode

Section 7.3 reports that the largest attribution is a metadata feature in 42% of the alerting windows and a missingness indicator in a further 23%, with time since admission to the ICU leading to 38% and the absence of a lactate result in 22%. Those features are not measurements of the patient, and this section states what follows, because the answer is not simply that the model is reading noise.

Temporal availability holds. Every metadata and missingness column is computed from observations, medication records and the start of the encounter at the end of the window *t*, and a window is labelled by an event in (t,t+12h]. Nothing in the vector is drawn from after the prediction time, and the generation of windows stops at the first event, so no feature can be contaminated by the treatment the event triggers. In the narrow question of whether the features precede the label, the design is sound.

Availability is not independence, and that is the real issue. Ordering an arterial blood gas or lactate is an act of clinical concern. If the bedside team suspects deterioration at 09:00, sends a lactate, and starts a vasopressor at 11:00, then the window at 09:05 carries a coverage indicator that is a record of the team’s suspicion, timestamped before the event and causally downstream of the same deterioration that defines the label. The splitting of patients in groups cannot eliminate this, because the mechanism operates within a single stay rather than across the division. The same argument  applies to the delay in documentation and the frequency of charting, both of which increase when a patient is closely watched.

Therefore, what the model predicts is better described as a composite: the onset of deterioration and the emerging recognition of it by the bedside team. For an early-warning system , these are not equivalent targets. A score that fires because the team has already sent a lactate is, to that extent, reporting a decision rather than anticipating one, and its clinical value is bounded by how much earlier it fires than the behaviour it is reading.

What it measures. The experiment refits feature subsets and compares: the full vector, the vector without the eight metadata columns, without the 21 missingness columns, without both and the complement, which is a care process with no physiological measurement available at all. Every subset is refit on the same patient-grouped split and scored on the same held-out patients. Table 14 reports it.

The result is larger than we expected. Twenty-nine columns containing no measurement of the patient reach AUROC 0.767, which against the full vector’s 0.815 is 85% of the discrimination the complete model achieves above chance. The deterioration in this cohort is substantially predictable from the timing of documentation, channel coverage, and measurements that were not taken, with no physical condition completely.

The two blocks are also largely redundant. Physiology alone reaches 0.808 and care process alone 0.767 while together they reach 0.815, so each recovers most of what the other carries, and removing either block alone costs nothing to three decimal places. That is the signature of two encodings of one underlying quantity, most plausibly the severity of the illness, which drives the physiology directly and the measurement behaviour through the bedside team.

Three consequences follow, and the second is one with practical force.

The care-process channel is real and large: a model can be built on this cohort that predicts the outcome nearly as well as the reported ensemble, while never seeing a vital sign or a laboratory value. Any claim that this system detects physiological deterioration must be read against that.

The channel is also removable at a negligible cost. The removal of all twenty-nine columns costs 0.007 AUROC, which is within the retraining spread of Section 7.8 and therefore is not reliably distinguishable from zero. A deployment concerned that its score may be reading clinician behaviour can eliminate the mechanism completely rather than reason about it, and give up nothing it can measure. We consider the physiology-only configuration as the more defensible one for a prospective study, because it makes the claim being tested unambiguous and we would run the trial on it.

What this does not establish is leakage, and we are careful about inference in both directions. Documentation intensity genuinely tracks severity: a deteriorating patient is measured more often for good clinical reasons, and a feature recording that is a legitimate severity marker rather than a contaminated one. The experiment cannot separate that from the clinician-recognition mechanism of the preceding paragraphs, because both produce the same correlation. The small drop on removal does not establish the absence of leakage, since physiological channels can carry the same information indirectly. Separating the two would require the time of the first clinician concerned. MIMIC-IV does not record it and we are unaware of a retrospective proxy that is not itself a care-process variable. What the experiment establishes is the size of the channel, which was previously unknown, and that it can be closed for 0.007 AUROC.

An additional observation is given in Section 6. The full-vector row here is the same quantity as the full ensemble of Table 5 on the same split, and the point estimates agree at 0.815 and 0.816; their intervals do not, the patient-clustered interval here spanning 0.025 against 0.006 for the window-level interval there. Clustered inference on every row of Table 5 is reported in Table 13, with width ratios of 3.6 to 3.9.

### 7.7. The Operating Threshold and the Alert Burden

The alert threshold is a property of a fitted model in a cohort, and up to this revision it was constant. config.RISK_THRESHOLD held 0.72, described as the $F_{1}$-maximizing value, and nothing derived it from anything. On a calibrated score at a 4.4% event rate, 0.72 asks for a better than even chance of deterioration inside twelve hours, which almost no window reaches: 621 of 160,748 held-out windows cleared it in the arm where we first measured this, catching 4.3% of positive windows and 12.9% of deteriorating stays. Every alert-burden figure computed at that threshold described a system that almost never speaks.

The ensemble now derives its threshold on the unresolved scores and stores it with the model. In the reported cohort, the maximum $F_{1}$ is τ=0.153, which captures 35.2% of positive windows with 27.9% precision. Table 15 gives that point and three sensitivity targets, because an early-warning system is specified by the fraction of events it must catch rather than by $F_{1}$.

The precision at a usable sensitivity is low: roughly 12 alerts per true positive at 90%. That is, there is a large amount of episode consolidation to address, and Table 16 measures it by replaying the policy of episode-level alerting in the waiting cohort.

Consolidation removes an order of magnitude. At 90% window-level sensitivity and 99% at the level of stay, the clinician receives 2.11 alerts per patient-day rather than 21.2. Expressed per eight-hour shift, the unit used in Table 17, that is, 0.70 against 7.07.

A figure of this order, roughly 0.8 alerts per patient per shift, is often quoted for systems of this kind, and it is reachable here: the episode-level figure at 90% sensitivity is 0.70 per eight-hour shift. But it is reachable only under both conditions together. The window-level figure at the same sensitivity is ten times it, and the window-level figure at the configured constant of 0.72 is fifteen times below it. Therefore, An alert burden and a sensitivity quoted together must come from the same operating point and the same unit of alerting, or the pair means nothing.

### 7.8. Transportability Across Admission Era

No external cohort was scored, and this is not a substitute for one. MIMIC-IV records anchor_year_group, which partitions admissions into three-year bands, so a model can be trained on the earlier bands and evaluated on the most recent. The site is unchanged, so this is not external validation. What it measures is transportability across practice change, documentation change, and case-mix drift over roughly a decade, which is the shift a system deployed next year actually meets.

Discrimination transfers. Against their in-distribution values in Table 5, the base learners move by at most 0.004 AUROC in the target band, in both directions, and the whole ensemble by 0.001, while the event rate increases from 4.4% to 6.0%, so the target is not just a resample of the source.

What the source-fitted row settles, and it is not what we expected.

Earlier versions of this table carried a confound we disclosed but could not remove: every ensemble row refits the meta-learner and the isotonic calibrator on the target band’s own labels, so those rows describe a site that has already recalibrated locally rather than one inheriting a trained system. The source-fitted row freezes the entire combination layer on the source and applies it unchanged, which is the inherited case. The two differ, but not in the quantity we had been cautioning about.

Discrimination is not affected. The frozen ensemble reaches 0.817 versus 0.815 for the target-refitted one, marginally higher and well within the run-to-run spread of Table 12. Refitting the combination layer on the target gave no ranking advantage at all, so the AUROC column of the ensemble rows was never inflated by the confound and can be read as stands.

Calibration is where the whole difference sits and the effect is large. Frozen, the ensemble’s ECE on the target band is 0.019; refitted, it is 0.000. For scale, 0.019 is what XGBoost alone achieves on that band without any isotonic stage at all, so what transport destroys is precisely the contribution of the calibration layer. The near-zero ECE reported for every other ensemble row in this table is therefore an artefact of refitting on the target, exactly as the earlier table note warned, and the honest figure for a site that inherits this system without recalibrating is 0.019.

This is the same conclusion Section 6.4 reaches from the threshold and Section 6 from the pipeline, arrived at by a third route: the ranking transfers between cohorts and the probability scale do not. A deployment may inherit the models; it must refit the calibration and re-derive the threshold on its own data.

How far a single run of this experiment can be read. The experiment was run four times from the same data and the same split, and Table 12 gives all four. XGBoost and the logistic comparator are identical at three decimal places in every run, as their seeds require. The deep learners are not: the LSTM returned 0.799, 0.805, 0.803 and 0.804, and the transformer 0.806, 0.811, 0.804 and 0.812, so retraining alone moves them by up to 0.008 AUROC. Every ensemble variant inherits the full ensemble ranging over 0.005. The bootstrap intervals in Table 18 do not cover any of it because they resample the evaluation windows while holding the fitted models fixed: they describe sampling error in the test set, not variance of the training procedure. No level in that table should be read to a precision finer than about 0.008, and the small movement against Table 5 quoted in the preceding paragraph is well inside that band, so the honest statement is that discrimination neither clearly decays nor clearly improves in absolute terms.

The leave-one-out margins are a different quantity, and they are more stable. Within one run the full ensemble and each reduced variant are built from the same base-model fits, differing only in which scores reach the meta-learner, so the margin between them is paired and the level’s variance does not enter it. Each run therefore supplies an independent estimate of each margin. All twelve estimates in Table 12 are positive and the ordering is the same in every run, which is the result that matters. The margins are not equally well determined: the transformer’s four estimates agree to within 0.003 and never fall below 0.007, while the LSTM’s span 0.001 to 0.005, so its contribution is consistently present and consistently small. Four retrainings are a small number and are not presented as a variance estimate, but it is the evidence that the question needs, and was not available from a single split.

One caution survives all this and no amount of rerunning will remove it: transport across a decade at one institution is the nearest thing the data in hand can offer, and it is not a substitute for transport across sites (Section 11.4).

### 7.9. Narrative Quality Assessment

The clinical utility of a generated narrative depends on three properties that standard NLP metrics do not capture: factual grounding, whether the narrative accurately reflects the data supplied; clinical coherence, whether it describes a picture a cardiologist would recognise; and actionability, whether the recommended action is the current standard of care for that presentation. ROUGE and BLEU evaluate surface overlap with reference texts and correlate poorly with clinical utility in medical NLP [74].

MedROAD V3 therefore specifies three scores for prospective assessment at the SERVIER deployment. The factual grounding score (FGS) is the fraction of factual claims traceable to a specific value in the vital-sign window, laboratory table, or SHAP attribution, assessed by a rule-based extractor with fuzzy matching for unit conversions and standard abbreviations; a narrative scoring below 0.90 is discarded in favour of the fallback template (Section 7.12). The clinical coherence score (CCS) comes from a secondary model invocation under a self-critique protocol [75]. The actionability score (ACS) rates the recommended action against the standard of care and requires a clinician rather than a model. Only FGS is computable automatically and only FGS is implemented and enforced at runtime, with the structural output contract checked before it; CCS and ACS require the prospective deployment.

What the grounding score does and does not establish. FGS is a lexical measure: extracts the numbers a narrative asserts and asks whether each traces to a value the prompt supplied, within a tolerance absorbing rounding and percentage restatement. A narrative that quotes every number correctly and then draws an unsafe conclusion from them scores 1.0, and the deterministic fallback of Section 7.14, which names a risk-lowering feature as a driver, passes it exactly this way. The illustration in Section 7.15 is a constructed example of the output contract rather than a generated narrative and is labelled as such; the two should not be read as the same artefact. The filter  therefore bounds the fabricated values and establishes nothing about clinical correctness; the implementation carries a test that asserts exactly this, so the score is not read as a safety guarantee. CCS, the dimension that would address clinical correctness, comes from the same family of self-critique models and is not an independent clinical assessor. Neither score substitutes for clinician review and the system does not treat them as doing so. The 0.90 threshold is a policy choice rather than a derived operating point: it permits roughly one unmatched number in a ten-number narrative, tolerating an incidental figure while rejecting a fabricated laboratory value. It has not been calibrated against human annotation, and the extractor has not been validated against human judgement of what constitutes a factual claim; both belong to the prospective study.

Evaluation status. Section 7.3 reports gate pass rates, the grounding distribution, and driver selection over 180 narratives generated from held-out cohort windows, and that measurement bounds what these safeguards have been shown to do. No hallucination rate is reported because setting requires clinician adjudication of the generated text. The narrative layer should accordingly be read as an implemented component whose runtime gates have been measured and found to be inert in this cohort, not as an empirically validated one. The model invoked is identified by the configured model string rather than fixed in the architecture, so the deployment record rather than this paper is the authority for which version produced any given narrative.

#### Evidence Carried over from MedROAD V2, and Its Limits

The published MedROAD V2 [10] reports a clinician evaluation of its language-model layer, and since V3 inherits the design of that layer, it is the only empirical evidence bearing on this component. It is set out here together with the reasons most of it does not transfer, the two systems asking the model to do different things.

Two board-certified physicians assessed 50 cases in V2. Diagnostic precision: the correct primary diagnosis appeared in 84% of the cases and the correct diagnosis within the top three of the differential in 94%. Citation accuracy, verified against the source literature: 89% accurate, 7% minor error with the correct paper slightly mischaracterised, and 4% hallucinated or incorrect. V2 further reports that making web-search verification mandatory for generated clinical content reduces overall hallucination from approximately 10% to approximately 3%, with fabricated citations falling by 85–95%, outdated information and guideline specifics by 60–70%, and drug dosing errors by 50–60%, at the cost of a three to five-fold latency increase.

Table 19 states what is carried, and V2’s headline numbers are not: V2 asks the model for a differential diagnosis grounded in the retrieved literature, whereas V3 asks for a three-sentence summary of a risk score and its attributions, with no retrieval, no citations and no diagnosis.

Three consequences follow, and the second is uncomfortable. The first is that V2’s largest measured hallucination category is structurally absent from V3, since removing retrieval removes fabricated citations.

The second is that V3 does not implement the main mitigation of V2. Mandatory web-search verification is what took V2 from roughly 10% to roughly 3%, and V3 has no equivalent, so for the categories that do transfer–mainly a recommended action and any dosing or guideline content implied by it–V3 sits in the unmitigated regime. It should not be assumed that V3 inherits V2’s 3% figure. Based on available evidence, it inherits the 10% regime for a narrower set of categories.

The third is that V3 has a mitigation that V2 lacks, with a precisely bounded scope. The filter for the factual basis rejects any narrative claiming a number that does not trace to a prompt field, which captures a fabricated dose, laboratory value, or vital sign, none of which would be supplied. It does not catch a drug named without a number, an inappropriate recommendation built from correctly quoted values, or an implied guideline claim that contains no figures. The filter and web-search verification are therefore complementary rather than alternatives: one bounds numeric fabrication, the other claims about the external literature.

Therefore, the evaluation of V2 does not discharge V3’s obligation to measure its own narrative layer, and is not presented as doing so. What it does supply is a protocol: two independent board-certified assessors over a bounded case series, rating defined dimensions against a reference standard, which the prospective study at the SERVIER deployment will follow. What that study adds to the runtime rates of Section 7.3 is the clinician adjudication that those rates cannot substitute for.

### 7.10. Governance of the External Model Call

Sending patient-derived values to a third-party inference endpoint is a health-information disclosure, and for a Canadian deployment it is governed by provincial health-information legislation and by PIPEDA. It is reasonable to regard this, rather than hallucination, as the more immediate barrier to clinical deployment.

Three measures follow. First, the patient identifier is not transmitted. The model is asked to write about a risk score and its drivers and never refers to the identifier, so sending it does not buy anything. Instead, A salted digest is sent, which keeps the alerts for a patient correlatable in the audit logs without the identifier leaving the deployment. This is pseudonymisation and not anonymisation: values remain health information about an identifiable individual wherever the key is held, so it reduces exposure without removing any obligation.

Second, prompt is restricted to what the task requires. The fields are numeric physiological values, their LOINC codes, and the SHAP magnitudes. The prompt does not have a free-text field, which is also what gives the structural immunity to prompt injection described below.

Third, and not resolvable in software: a deployment transmitting these values requires a data-processing agreement with the model provider covering permitted use, retention, and sub-processing, confirmation of the jurisdiction in which inference is performed and whether any payload is retained or used for training, and an institutional privacy impact assessment under the applicable provincial statute. We state these as prerequisites rather than as completed work, and none of them was in place for the work reported here. They are prerequisites for a deployment rather than for this evaluation: the 180 narratives of Section 7.3 were generated from MIMIC-IV, a research extract de-identified under its own data use agreement, so no personal health information was disclosed in producing them. A live deployment sends values about an identifiable patient, and the position is therefore different in kind, not in degree. A site unwilling or unable to meet these requirements can run the system with narrative generation disabled, in in which case every alert is served by the deterministic template, and no patient-derived value leaves the deployment.

### 7.11. Hallucination Mitigation and Fallback

Hallucination, the generation of plausible but factually incorrect clinical content , is the main safety risk of integrating a language model into a decision support system [43,76]. Four mitigations operate together. The prompt provides all the numerical values that the narrative is expected to reference and instructs the model to introduce nothing further, the retrieval-augmented pattern that substantially reduces hallucination in clinical settings [77]. The sampling temperature is set to 0.2, well below the default, trading linguistic variety for consistency, which is the correct trade in this context. The FGS filter rejects any narrative asserting a number that cannot be traced to a prompt field, which bounds fabrication but not misinterpretation. A fifth mitigation available for this design is not implemented: published MedROAD V2 [10] reports that making web-search verification mandatory for generated clinical content reduces its overall hallucination rate from roughly 10% to roughly 3%, at a cost of three to five times latency. V3 does not perform retrieval, so for error categories that are transferred between the two systems, it operates in the unmitigated regime. Finally, the output contract is validated structurally before write-back, so a response deviating from the expected three-sentence form, naming the system’s own nature, or carrying a demographic descriptor, is diverted to the fallback rather than reaching the chart.

### 7.12. Fallback Narrative Template

When the Claude API is unavailable, is thrown out, or returns a narrative that fails the FGS threshold or structural check, a deterministic template is substituted. It is populated directly from the risk score and the top two SHAP features, producing text of the same three-sentence shape without any generative component. The system therefore degrades to a slightly less fluent alert rather than to no alert at all, which matters because the fallback path is exercised precisely when something has already gone wrong. Because the template is deterministic, its output is auditable and its latency is negligible, removing the 1.8 s API term from the critical path.

### 7.13. Adversarial Input and Fairness Safeguards

Free-text EHR fields are an untrusted input channel: a note containing instruction-like text could, in principle, redirect the model’s output [78]. MedROAD V3 avoids this class of attack structurally rather than by filtering. No free-text field is ever placed on the prompt. The user prompt is assembled exclusively from numeric values, LOINC codes, and SHAP magnitudes drawn from the feature vector, so there is no pathway by which text authored by a third party reaches the model. The system prompt is fixed at deployment and never templated from runtime data, and the output contract is validated before write-back, so a response that deviates from the expected three-sentence structure is rejected in favour of the fallback template.

Fairness belongs to the ensemble rather than to the narrative layer, since the narrative is conditioned on the risk score and its SHAP attribution and inherits any bias present in them. Narrative text is checked for demographic descriptors, which have no legitimate role in a three-sentence summary conditioned on physiology; an occurrence diverts the alert to the fallback template and is logged. Stratified monitoring of alert rate, positive predictive value, and calibration error by age band, sex, and recorded ethnicity, with recalibration triggered by sustained divergence between strata according to the procedure of Section 6.3, is specified but not implemented in the system evaluated here, and no stratified results are reported. It is a requirement of the prospective deployment rather than a property of the present work.

### 7.14. Worked Example: The Prompt as Emitted

The Listing 3 is a complete user prompt as the evaluated implementation emits it, for a window with R=0.843. The assembly is genuine: it is the output of the prompt-assembly code, which is deterministic and does not require an API call, so it can be reproduced exactly from the distributed source. Its inputs are not a cohort window. The risk score and the five attributions were specified by hand to represent a plausible deterioration case, so the listing demonstrates the prompt’s structure rather than reporting a measured explanation, and the fifth label, “obs count”, does not correspond to any column of Table 10. Regenerating the instance from a held-out window of the evaluation cohort would remove both qualifications.
**Listing 3.** User prompt instance as emitted by the evaluated implementation (*R* = 0.843). Compare
with the specified template of Listing 2.Case reference: pt-c5ec64213962 Ensemble risk score: 0.843 (threshold 0.72) Top physiological drivers (SHAP analysis):  1. systolic bp rate (reduced, SHAP = -0.412)  2. lactate (elevated, SHAP = +0.383)  3. heart rate mean (elevated, SHAP = +0.221)  4. bnp (elevated, SHAP =+0.164)  5. obs count (reduced, SHAP = -0.071) Write the three-sentence clinical alert.

Three properties of this instance are worth considering. The case reference is a salted SHA-256 digest truncated to twelve hexadecimal characters, and no element of the prompt identifies the patient or the institution. The names of the internal characteristics are rewritten in clinical labels using a substitution table, so vital _systolic _bp _rate is presented as the “systolic bp rate”, which is readable but not idiomatic and is the coarsest part of the pipeline. The Shapley value is supplied along a discretised direction, giving the model both the magnitude it may quote and the sign it must respect; a narrative describing a negative-attribution feature as elevated would be factually wrong in a way the lexical grounding score cannot detect since the number itself would match.

Against this prompt, the model returns:

*Patient* pt-c5ec64213962 has a risk score of 0.843, exceeding the alert threshold of 0.72 and indicating a high risk of clinical deterioration. The leading contributors to this elevated risk are elevated lactate and an elevated mean heart rate. Perform an urgent bedside assessment for tissue hypoperfusion, including a repeat lactate measurement.

This response passes the output contract at three sentences and 347 characters, and scores 1.000 on the factual grounding measure as that measure now stands: the two numbers it asserts, 0.843 and 0.72, are both values the prompt supplied. Under the first implementation of the check it scored lower, for reasons taken up at the end of this section.

It also illustrates a selection question that neither gate addresses. The system prompt asks for the two most important drivers. The response names lactate at ϕ=+0.383 and mean heart rate at ϕ=+0.221, the two largest positive attributions, and omits the systolic pressure rate, which has the largest magnitude of the five but a negative attribution, ϕ=−0.412. A negative Shapley value lowers the predicted risk: in this window the systolic trajectory is evidence against deterioration, not for it. For a narrative explaining why the risk is elevated, leaving it out is the correct selection, and the recommended action, an assessment for hypoperfusion with a repeat lactate, follows from the drivers the narrative actually names.

The weakness is in the prompt rather than in the model. The prompt lists all five attributions under the heading “Top physiological drivers”, so a feature that lowers the risk is presented as a driver of it, and the word “reduced” encodes the sign of the attribution, which a reader can easily take for the direction of the measurement: “systolic bp rate (reduced)” reads as a falling pressure, which is not what ϕ<0 reports. The model resolved the ambiguity correctly here, but nothing in the prompt makes it do so. Risk-raising and risk-lowering attributions should be listed separately, the ranking the narrative is asked to respect should be stated as one over the risk-raising attributions, and the direction word should describe the measurement rather than the attribution, which is what the corrected formatter of Section 7.3 now does.

This is the limitation the grounding measure declares, now instantiated: the score bounds fabrication and says nothing about whether the right facts were selected. A narrative that quotes supplied values correctly but names a risk-lowering feature as a driver passes both gates, which argues that the narrative evaluation deferred in Section 7.9 must test driver selection against the sign as well as the magnitude of the attributions, rather than only grounding.

With the API unavailable, the deterministic fallback instead produces:

*MedROAD* V3 has assigned an elevated risk score of deterioration of 0.843, exceeding the optimal clinical threshold of F1 of 0.72. The primary physiological drivers are the systolic bp rate (reduced, SHAP = −0.412) and lactate (elevated, SHAP = +0.383). An immediate clinical evaluation at the bedside is recommended.

The fallback ranks drivers by magnitude without regard to sign, so it names the systolic pressure rate, the one feature in the list that lowers the risk, as a primary driver of an elevated score, and both gates pass it. That is the selection error in this example, and it sits in the deterministic path rather than in the model; the template should draw its drivers from the risk-raising attributions only.

In the first implementation of the grounding check, both of these texts exposed false-rejection defects and both would have been rejected by the filter that was supposed to protect them. The fallback scored 0.750 because the alert threshold, which the prompt states and which the first system-prompt instruction invites the model to quote, was not in the set of supplied values, so a correct quotation counted as fabrication. The response of The model scored 0.333 rather than the 1.000 it receives now, because the pseudonymized case reference it echoed was parsed as clinical assertions: the number pattern matched at most three digits at a time, so pt-c5ec64213962 contributed 5, 642, 139 and 62 as unmatched values. Nothing in the prompt tells the model not to repeat the reference, and the a twelve-character digest will usually contain digit runs, so this would have rejected a large share of well-formed narrations.

Both are fixed: the threshold is a supplied value, and the digit runs inside an alphanumeric token are no longer read as assertions, which also excludes clinical tokens such as SpO_2_ and B12. Both behaviours are pinned by tests. We report them because they are an indicator of how any acceptance rate for this filter should be read. A grounding filter suppresses correct narratives as easily as fabricated ones, and until its false-rejection rate is measured against annotated output, a high rejection rate cannot be distinguished from a model that performs badly. Ours was rejecting both of the only two narratives we had.

### 7.15. Example Narrative Output

Table 20 illustrates the intended form of a MedROAD V3 narrative for a constructed case with R=0.84, along with the input values from which it would be derived. It is an illustration of the output contract rather than a generated sample; narrative quality is prospectively assessed as described above.

## 8. Write-Back to OpenEMR

MedROAD V3 returns its output to the patient chart over two different interfaces, and which one a deployment uses is decided by the server rather than by preference. This section describes both, beginning with the one that runs on the reference deployment, since the FHIR path does not work there.

### 8.1. The Deployed Path: OpenEMR’s Standard REST API

OpenEMR exposes two independent HTTP interfaces with separate OAuth2 scope families: an FHIR R4 API under api:fhir, and its own REST API under api:oemr. They differ in what they permit. Of the 181 scopes OpenEMR 7.0.2 advertises, .write exist for only three FHIR resources, none of them clinical; the standard REST API offers write scopes for soap_note, medical_problem, message, document and transaction, among others. The Write-back to this server therefore goes over the standard REST API, not over FHIR.

The alert and its narrative are written as a SOAP note on the encounter, using the route of creation exposed by this release (POST /api/patient/:pid/encounter/:eid/soap_note). The mapping of the SOAP structure is shown in Table 21. The destination suits the content better than the original design did: a SOAP note is the native clinical note a bedside physician reads, whereas a DocumentReference is a pointer to an attachment.

**Figure 3:**
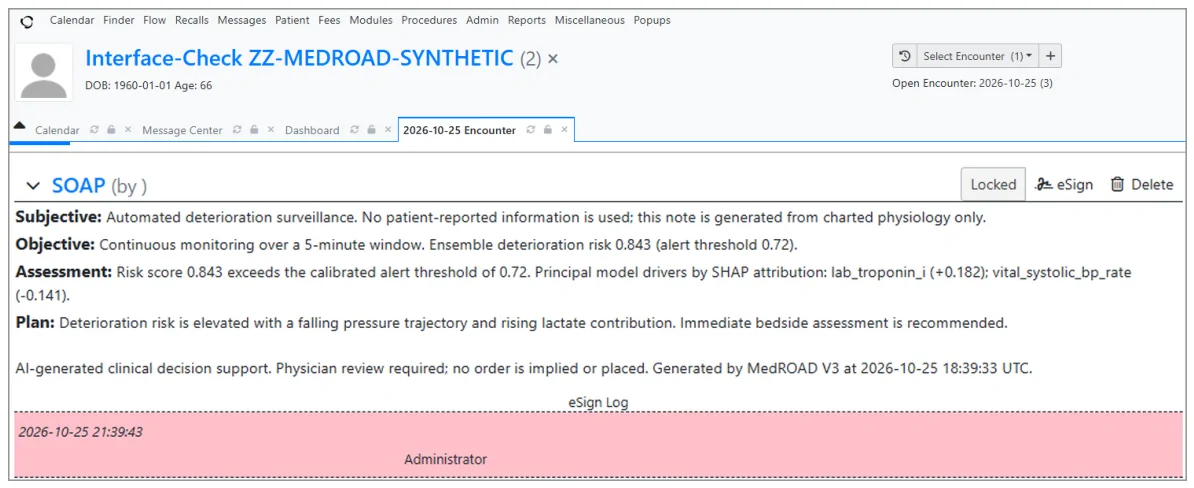
Alert and narrative delivered to the patient chart over OpenEMR’s standard REST API and displayed in the encounter view of OpenEMR 7.0.2 (localhost:8300). The risk score and the threshold shown are the interface-check harness’s fixed demonstration values, which predate threshold derivation and are not the operating point of Section 7.7; the figure demonstrates the write path and the note’s structure, not a model decision.

Figure 3 shows the result in the graph: a note written by the interface-check harness through this route, rendered by OpenEMR’s own encounter view with no modification to the server. The four sections carry the content specified by Table 21, the provenance statement closes the plan, and the note is locked and attributed in the eSign log. The patient is synthetic and is named to make that unmistakable in a chart; the harness refuses any non-localhost host without an explicit override, so a demonstration write cannot reach a live server by accident.

The path has been exercised from end to end against a live instance. The distributed scripts/probe_writeback.py acquires a token under the standard-API scope family, creates a synthetic patient and an encounter, and writes the SOAP note carrying the risk score, its SHAP drivers and the generated narrative. The encounter creation illustrates why the writer discovers required fields rather than hard-coding them: the server rejected the first attempt, naming pc_catid and class_code as required, and the write succeeded on the second with those added.

The provenance is carried out in the note’s own text rather than in a structured field. This is a loss relative to the FHIR design, in which an Extension records the authoring system, model version, inference timestamp and risk score in machine-readable form and supports retrospective audit queries. A SOAP note does not offer such an extension point, so the same facts are stated in prose, which a human auditor can read, and a query cannot aggregate. A deployment with audit obligations that require structured provenance should weigh this.

Two further consequences follow, and we state them rather than leave them implicit. First, this path is vendor-specific. It integrates with OpenEMR in particular and writes OpenEMR’s own records, not FHIR resources, so a deployment using it is not standards-integrated in the sense the architecture otherwise claims, and the write-back component does not travel to another EHR unchanged. The event-source, streaming, feature, and inference layers remain standards-based; only the final write is vendor-coupled. Second, of the four types of resource the write-back client emits (Table 22), only the narrative has a confirmed creation route in this release. There are scopes for resources whose creation routes we have not verified, and a scope without a route is not usable, so the risk-alert flag, the escalation request, and the proposed order are not currently written on this server.

### 8.2. The Portable Path: FHIR Resources

Where a server supports it, FHIR is the preferable target: it is standards-native, carries structured provenance, and travels between EHRs without modification. MedROAD V3 implements this path, and it is the design around which the architecture is built; it is simply unavailable in OpenEMR 7.0.2. Table 22 gives the mapping.

All resources carry a provenance Extension that identifies the authoring system, the model version, the inference timestamp and the risk score, allowing retrospective audit queries.

ServiceRequest resources are created with status=proposed and intent=proposal, routing to order entry as draft orders requiring explicit physician sign-off. MedROAD V3 never creates autonomous active orders, consistent with the human-in-the-loop design principle discussed in Section 10. The same constraint is carried in the SOAP note of the deployed path, which states that no order is implied or placed.

Flag resources are managed by upsert: before creating a Flag, the client queries for an existing active Flag for the patient and updates it via PUT rather than creating a duplicate. When *R* falls below τ the Flag is patched to status=inactive, providing an explicit resolution event in the audit trail. The path of The deployed SOAP-note does not have an equivalent resolution mechanism, since the note once written is not retracted. Therefore, a resolved alert is visible in the FHIR design and not in the deployed one.

### 8.3. Choosing Between Them

A deployment should empirically determine which destinations its server accepts rather than assume either path. The three requirements are independent and fail separately: the resource type must exist, it must expose a creation interaction, and a write scope must be obtainable for it. The conformance tooling distributed with this work reports all three per destination for both interfaces.

## 9. Evaluation

### 9.1. Cohort and Experimental Protocol

The evaluation uses MIMIC-IV v3.1, restricted to admissions whose first ICU unit was the Coronary Care Unit or the Cardiac Vascular Intensive Care Unit, with a minimum stay of six hours, with events in the first six hours suppressed as Section 9.2 sets out. This yields 25,214 eligible stays, of which 5907 (23.4%) carry a deterioration event under the composite definition of Section 5, giving 1.75 million five-minute inference windows at a window-level event rate of 4.4%. The experiments report the full cohort rather than a subsample.

#### Label Construction and Censoring

The temporal treatment of windows around an event determines what learning problem the 1.75 million windows actually pose, so it is stated in full.

Which agents count as an event. The composite outcome counts initiation of norepinephrine, epinephrine, vasopressin, dopamine or dobutamine. Phenylephrine and milrinone are excluded, and because this choice materially shapes the label, we give the reasoning rather than the list alone.

Phenylephrine is a pure $\alpha_{1}$ agonist without inotropic action, given in this population as intermittent boluses or short infusions for transient hypotension: after induction of anaesthesia, during sedation, or around a procedure. In a cardiac cohort, it is ordered for a large majority of patients at some point, and its administration marks a brief pressure excursion rather than a circulatory failure. Milrinone is a phosphodiesterase-3 inhibitor used as an inodilator, characteristically started electively in advanced heart failure, as a bridge to transplant or device, or continued before admission; its initiation often reflects a planned change in chronic therapy rather than acute decompensation. Counting either as deterioration would make the label a marker of hemodynamic support of any kind, which is a materially different and far more prevalent clinical question than the one this system addresses.

Two points of honesty are attached to this. The decision is a modelling choice, not data cleaning, and it moves the event rate substantially rather than marginally. And it is not self-evidently correct: a reader may hold that the initiation of milrinone in a patient who has not previously received it is exactly the decompensation worth predicting. The extraction tool therefore accepts a --marginal-vasopressors flag that rebuilds the cohort with both agents counted. That analysis has now been run at the level of the cohort and is reported in Section 9.2, which also reports a larger problem that running it exposed.

Only the first deterioration event is used in a stay. A window beginning at *t* is labelled positive when that event falls into (t,t+12h] and negative otherwise. The window generation for a stay stops at min(ICUdischarge,firstevent), so no window is generated after an event at all.

Four consequences follow, answering the questions this design raises. All windows within twelve hours of the first event are positive. The windows after it are censored rather than labelled, because the treatment the event triggers, a vasopressor infusion or an endotracheal tube, is recorded in the same tables as the physiology, and a model allowed to see would learn to recognise the response rather than predict the event. A patient contributes at most one positive episode: no second vasopressor initiation is achieved later in the same stay. And no window is opened after a vasopressor has started, so the question of whether it remains positive does not arise.

The cost of this choice is that the model is trained to anticipate a first deterioration in a patient not yet being treated for one and says nothing about recurrent deterioration in a patient who already receives support. That is the clinically useful question for an early-warning system, but it is narrower than the composite outcome’s wording alone suggests, and a deployment should not expect the score to follow an established episode.

### 9.2. Sensitivity of the Label to the Outcome Definition

The composite label consists of three choices: which care units enter the cohort, how long after admission events to the ICU are suppressed, and which agents count as vasopressors. Each was made deliberately and documented above. None had been measured. Table 23 measures them, by rebuilding the MIMIC-IV v3.1 cohort under each combination.

The blanking window dominates, and one hour is too short. Holding the full cardiac cohort fixed and moving the blanking window from one hour to six removes 9449 of the 11,677 ventilation events. Patients admitted from the theatre are intubated, and therefore a one-hour window labels the timing of a scheduled extubation as a deterioration event. At one hour, ventilation alone marks 46% of the 25,214 stays.

The surgical unit is not the problem. Our first reading of this was that the Cardiac Vascular Intensive Care Unit should be excluded, since dropping it in a one-hour window removes 10,344 ventilation events against 787 for the window change. That attribution is wrong, and it is wrong in an instructive way: it varies one factor at a time across a pair that interacts strongly. Once the window is six hours, dropping the surgical unit removes only 1682 additional events, and the two units are then indistinguishable in event rate, 23.9% in CVICU against 23.6% in CCU. The full cardiac cohort with a six-hour window is therefore preferable to a halved one: it retains 25,214 stays in the same label quality.

The same rate means different things in the two units. CVICU mortality is 3.2% compared to 9.5% in CCU, and initiation of vasopressors accounts for 72% of CVICU events compared to 79% in CCU with far more deaths behind the remainder. The pooled discrimination therefore averages two clinically distinct problems, and a breakdown per-unit should accompany any pooled figure.

The marginal agents more than double the stay-level rate. Counting phenylephrine and milrinone takes the one-hour cohort from a rate we did not complete to 62.2%, labelling nearly two thirds of all stays in the cardiac ICU as deteriorating. This supports the exclusion argued above, and the extraction tool now refuses to pass silently: it warns when the rate exceeds 35%.

### 9.3. The Reported Cohort and Its Partitions

Each result in this paper is drawn on a label, referred to throughout as the reported cohort: the full cardiac units with a six-hour post-admission blanking window, giving 5907 of 25,214 stays affected (23.4%) and 1.75 million five-minute windows at a window-level event rate of 4.4%. Two properties of this label are consequential enough to state here.

The first is the blanking window, which is distinct from the six-hour minimum length of stay required for a stay to enter the cohort; the two filters are easily conflated and have different effects. Blanking at six hours rather than one excludes post-operative ventilation from the event set. That exclusion matters because a post-surgical admission is predictable from the first window of a stay, from admission context rather than physiology, so discrimination measured against a label that counts it is, in part, the identification of surgical patients. Section 9.2 quantifies how far the label moves under the alternative definitions.

The second is a limitation on reproducing the label exactly. The labels behind the reported results were produced by a version of the extraction script that is not the one distributed with this work, so they cannot be regenerated bit by bit from the released tool, and a reader who reproduces the pipeline should expect small differences rather than be surprised by them. Separately, and for a reason that is definitional rather than a defect, the modelling cohort carries 5907 events against the 6000 in the six-hour row of Table 23: window generation emits nothing for a stay with no charted observation in any five-minute interval before its event or discharge, and 93 stays meet that description. The two numbers count different things and neither supersedes the other.

Each segment is grouped by patient. Consecutive five-minute windows from one stay are near-identical, so a split drawn over windows places the same individual on both sides and the model learns to recognise patients rather than deterioration; the inflation this produces is large enough to be mistaken for a result. Grouping is enforced programmatically and training aborts if any patient appears on both sides of a split. The three-way ablation of Section 6 draws its own grouped partition of 15,206 training and 5049 held-out patients, the latter supplying the 435,307 evaluation windows of Table 5. The saved model used for the threshold and alert-burden analyses carries a separate partition whose hold-out side is 3514 stays and 160,748 windows.

Out-of-fold predictions for the meta-learner are generated under grouped five-fold cross-validation on the training and validation partition, so the meta-learner never sees a patient on whose labels its inputs were fitted. The fitted meta-learner assigns a comparable weight to all three base models (XGBoost 2.50, LSTM 2.14, Transformer 2.38, intercept −5.00), consistent with the ablation finding that each contributes.

### 9.4. Latency Characterisation

Latency is reported in two parts because only one of them can be measured without a live deployment. The compute path includes feature assembly, the three base models, the meta-learner and SHAP attribution, and is measured directly on windows drawn from the evaluation cohort. The transport path includes FHIR delivery, Kafka produce and consume, and write-back to the chart, which depends on infrastructure and is reported separately against a running stack.

Measurements are percentiles of repeated single-window invocations after warm-up, not means. A per-patient alert is one window’s work, so the single-window figure is the operative one, and for a real-time guarantee, the tail rather than the average determines whether the deadline is met.

The compute path completes in 3.09 ms at the median and 3.72 ms at the 99th percentile. The per-stage estimates carried in the published MedROAD V2 architecture [10] allowed 60 ms for ensemble inference and a further 5 ms for SHAP attribution, which is the same work the compute path performs here, so the comparison is 65 ms estimated against 3.09 ms measured. The three base models and the meta-learner alone account for 1.81 ms of that, and feature assembly, which V2 budgeted for separately under window aggregation, for a further 0.22 ms. The discrepancy is not a tuning result. Those estimates were made for a design in which each inference issued a network call to a model server, whereas the present implementation holds all three models resident in the stream processor, so the inference cost is a function call rather than a round trip.

Each row of Table 24 is timed in its own loop over independently sampled windows, and the total row is timed in a further loop that runs the path end to end with SHAP attribution gated on the alert threshold exactly as it is gated in deployment. The rows are therefore separate measurements of one pipeline rather than a decomposition of a single measurement, and the total is not the sum of the rows above it: it excludes feature assembly, which is timed through the feature engineer rather than the pre-built matrix, and charges SHAP only on the windows that cross the threshold. The total is the figure to carry forward, since it is the latency a single alert actually incurs.

The practical consequence is that computation is not the bottleneck, and tightening it further would be a wasted effort. Table 25 sets the measured compute figure alongside the remaining stages: the budget is dominated entirely by transport, and specifically by the two FHIR round trips at either end.

The two transport stages that MedROAD controls were measured against the running deployment (Table 26): 3.1 ms at the median from webhook receipt to Kafka acknowledgement, and 0.8 ms for a direct Kafka produce. These are one to two orders of magnitude below the architectural estimates they replace, which allowed 180 ms for webhook delivery and 80 ms for Kafka ingestion. The estimates were made for a design in which each hop crossed a network boundary; co-located services reduce both to a few milliseconds. The webhook’s p99 of 25.8 ms against a p50 of 3.1 ms reflects occasional Kafka metadata refreshes, and is the only tail of consequence in the measured path.

Three cautions are attached to this table. The first row is not a latency but a property of the event source: under Subscription it is the delivery time, under polling it is bounded below by the poll interval, and in the OpenEMR deployment described here, it therefore dominates everything beneath it. The write-back row (‡) is now measured rather than estimated. No FHIR write can be timed against OpenEMR 7.0.2, which offers no .write scope for any FHIR clinical resource, but the standard-API path that runs there (Section 8.1) completes a SOAP note POST in 33 and 35 ms on two runs against a local instance, including the field-discovery retry the encounter creation requires. Two observations are not a distribution, and unlike the compute rows, this figure is not a percentile over repeated invocations; it is quoted as a range for that reason. It is also a single-host figure with no network between the client and the server, so a deployment across a hospital network should expect more. And the narrative term, which dominates the alerting path by a factor of twenty, is third-party API latency outside the system’s control—which is why narrative generation is conditioned on the alert threshold and backed by the deterministic fallback of Section 7.12 rather than placed on the critical path.

Taken together, the two measured stages between receiving an observation and emitting a score—webhook ingestion and the compute path—account for roughly 6 ms, which is negligible against any clinical timescale. The unmeasured stage between them is not: the Faust consumer-group poll is estimated at 50 ms and is the largest term MedROAD itself owns, larger than the 33–35 ms write-back. The honest reading of the table is therefore that MedROAD’s measured computation is free, that its one unmeasured internal stage dominates its own contribution and should be instrumented before any end-to-end claim is made, and that the outer bounds are set by the EHR’s event delivery at one end and its write-back at the other. The Claude API invocation adds 1.8 s but is triggered conditionally (R≥τ), so that latency is incurred only on alerting windows.

### 9.5. Scaling with Concurrent Patient Count

**Figure 4:**
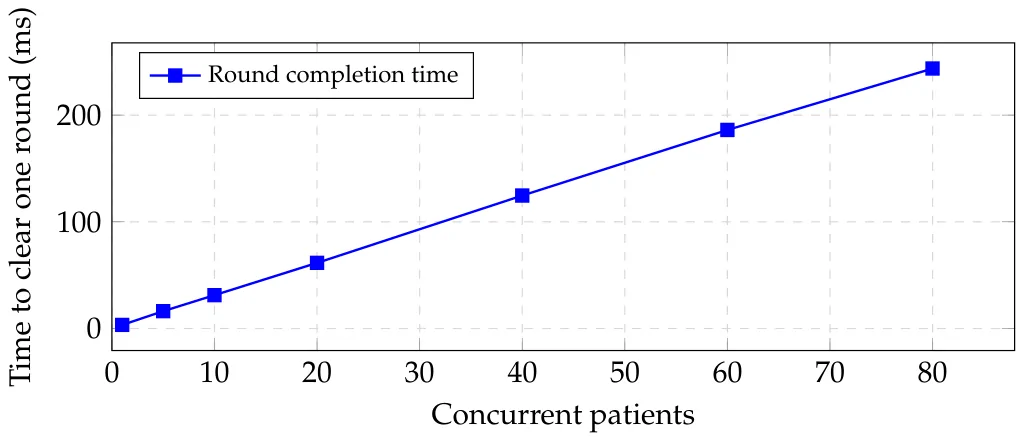
Compute-path scaling. Each round scores one five-minute window per concurrent patient through the full inference path. Transport is excluded.

The operational question is whether the system stays up: with *N* monitored patients each producing a five-minute window, each window must be scored before the next arrival. That imposes a hard deadline of 300 s per round and defines a saturation point beyond which backlog grows without bound. Figure 4 sweeps *N* from 1 to 80.

Scaling is linear and the constant is small. At 80 concurrent patients, a round is completed in 244 ms, consuming 0.08% of the 300 s window period. The median Per-window latency is flat across the sweep, declining slightly from 3.24 ms at N=1 to 2.98 ms at N=80 as the caches warm, indicating that there is no contention over this range.

The practical reading is that inference capacity is not a design constraint in any plausible cardiac ICU size. A 40-bed unit uses less than 0.05% of the available window period, and linear extrapolation places saturation of the compute path far beyond any single-site deployment. This measurement covers only the compute path; broker and FHIR transport under concurrent load are not included, and according to the evidence of Table 25 they, rather than inference, determine the achievable patient count.

### 9.6. Comparison with Rule-Based Early Warning Scores

Claims about alert burden are only meaningful at a matched operating point because any system reduces its alert count by detecting fewer. NEWS and MEWS were therefore computed from the same engineered feature vector, de-normalised to clinical units, and each system was evaluated at thresholds achieving the same sensitivity. All three are scored in the patients held during training and Table 17 reports the result.

On the choice of comparator. NEWS and MEWS are the scores that a cardiac ICU would otherwise be running, which is why they appear here: the comparison answers what a site gains by replacing its current instrument. It does not establish that the ensemble is the best available model for this cohort, and it is not evidence of architectural merit, since a simpler model consuming the same features might well match it. The informative comparators for that question are a regularised logistic regression and a gradient-boosted model trained on the identical group split, and the published MedROAD V2 scored on the same waiting patients. The first two are reported in Table 5: the logistic model reaches 0.758 and XGBoost alone 0.805, against 0.816 for the ensemble. The third is absent and its absence is a real limitation rather than an oversight of the presentation. Against the rule-based scores alone, the comparison would demonstrate domain mismatch at least as clearly as model superiority; it is the same-split linear and single-model rows, not the NEWS and MEWS columns, that establish the ensemble earns its complexity.

At at every operating point, MedROAD V3 generates between 39% and 64% fewer alerts than the rule-based scores while detecting the same fraction of events. The margin is widest at 70% sensitivity and narrowest against NEWS at 90%, where the ensemble has to reach far enough down its own distribution that much of its advantage erodes. The discrimination gap is the more durable result: NEWS at 0.590 and MEWS at 0.578 are close to chance in this cohort.

The ensemble’s 0.826 in this table is not the 0.816 in Table 5, and the difference is one of the evaluation set rather than of the model. The ablation refits every variant on its own grouped split and scores 435,307 holdout windows. This comparison scores the single saved ensemble on the 160,748 windows belonging to the patients named in that model’s split file, which is also the cohort of Table 16. The heading figure quoted elsewhere in this paper is 0.816, because is derived from the larger evaluation and from the procedure used for every other variant. The value 0.826 is reported here so that the AUROC column and the alert-rate columns describe the same windows.

That result deserves attention rather than celebration. NEWS and MEWS were developed and validated for patients in the general ward, where the task is to detect deterioration in a population that is mostly stable. A cardiac ICU population is already selected for instability, is already continuously monitored, and is already receiving the interventions these scores exist to trigger. Their near-chance performance here is evidence that they are being applied outside their intended setting, not that they are poor instruments. The fair reading is that a score trained in the deployment population substantially outperforms a generic one, which is an argument for site-specific modelling rather than against early warning scores.

The second caveat concerns absolute numbers, and is in part an artefact of what is being counted. Alert rates of 22 to 50 per patient per shift are far too high for clinical use. They follow from fixing the sensitivity at 70% or higher in a population with a window-level event rate of 4.4%, and a deployable configuration would accept a lower sensitivity for a workable burden. But they are also counts of windows above threshold rather than of clinical events: the scorer runs every five minutes, and in the system evaluated, each positive window was an independent alert. Therefore, A single two-hour deterioration episode contributed 24 alerts.

#### Episode-Level Alerting

Counting windows is the wrong unit for a burden figure, and the absence of any consolidation was a genuine gap rather than a reporting convention. The implementation now incorporates an alerting policy between the scorer and the clinician, which converts window-level decisions into episode-level ones. The first window above the threshold opens an episode and alerts. Subsequent windows in the same episode do not, however, there are. An episode whose risk climbs materially above the value that opened it alerts again because a patient deteriorating after an unacknowledged alert is new information and that escalation itself is rate limited so that a steadily rising score cannot ladder into a stream of notifications. An acknowledger of an episode suppresses it until it resolves or escalates. The risk below the threshold for a sustained period closes the episode, with the delay chosen so that a score oscillating around the threshold does not open a new episode at every crossing.

Two points of discipline are attached to this. The policy changes no prediction: discrimination and calibration are properties of the scorer and are untouched, so an alert-burden reduction obtained this way must not be presented as a modelling improvement. And the figures in Table 17 are the window-level counts, which are what the comparison with NEWS and MEWS measures because neither rule-based score has an episode policy to replay and consolidating only the ensemble’s alerts would flatter it. The episode-level burden is quantified separately, by replaying the locked windows through the policy, in Table 16.

## 10. Regulatory Considerations

This section offers a preliminary regulatory analysis, not a classification. The class of devices according to the SaMD guidelines of Health Canada [55] and the IMDRF risk-based framework [56] is determined by the regulator based on a proposed use statement submitted, and nothing here substitutes for this determination. We set out our reading, and then the argument against it because the second is substantial.

The considerations that would support Class II (moderate risk) are:

- The purpose is to inform clinical decision-making rather than drive it, with incorrect output resulting in delayed rather than denied treatment.
- All AI-generated outputs are explicitly labelled and require the physician to review before action; no autonomous orders are generated.
- The system does not directly control therapeutic devices.

The considerations pointing to a higher class deserve equal weight, and are recorded rather than resolved. The predicted outcomes are vasopressor initiation, invasive mechanical ventilation, and ICU death, which are life-threatening in any ordinary reading, and the IMDRF framework assigns a class based on the severity of the healthcare situation, as well as the importance of information. The narrative layer may name a specific agent, as in the worked example in Section 7.15, which moves it closer to driving a decision than to informing one. Human oversight does not settle the class by itself either: a requirement for physician review is a mitigation, not a reclassification, and regulators have declined to treat it as one. The balance is genuinely uncertain and the question must be settled by formal classification against a submitted intended-use statement. A reader should treat the Class II reading as a hypothesis the authors hold, not as a classification the system has.

However, three architectural decisions were made with the requirements of Class II in mind, and would in any case be required in a higher class. The SHAP explainability layer provides the instance-level documentation that audit-trail compliance requires. Resources ServiceRequest are created with intent=proposal rather than order, keeping the human in the loop by construction. And Kafka events are retained for 30 days, with FHIR write-back resources carrying provenance extensions and the SOAP note of the deployed path stating the same facts in its text, enabling the retrospective audit of Health Canada’s post-market surveillance obligations require.

As documented by Muehlematter et al. [57] and Wu et al. [58], prospective clinical validation is a consistent weakness of approved AI medical devices. The planned deployment at the SERVIER Virtual Cardiac Centre will include a prospective non-inferiority study comparing MedROAD V3-assisted care with standard NEWS-based monitoring, with primary endpoints of rapid response team activation rate, false alert rate, and time-to-intervention for cardiac deterioration events.

## 11. Discussion

### 11.1. Telemedicine and Remote Patient Monitoring Applications

The sensor-agnostic design of the FHIR event source layer has direct implications for the deployment of telemedicine.

Three cases of chronic disease use illustrate the breadth of applicability. Post-discharge heart failure monitoring is the most immediate: daily weight, SpO_2_, resting heart rate, and blood pressure, scored by an ensemble trained in outpatient observations, would produce a calibrated readmission risk daily and alert a nurse navigator only above threshold, addressing the alert fatigue that characterises current RPM nurse workflows. Continuous glucose monitoring fits directly into the existing Faust window processor, since a CGM is published at five-minute intervals; Laboratory elements would be replaced by statistics of glycaemic variability such as time-in-range and nocturnal hypoglycemic index, and the LSTM branch is well suited to the autoregressive dynamics of the glycaemic series. Ambulatory arrhythmia surveillance would route rhythm classifications published under LOINC 8884-9 through the same broker, with the Transformer branch trained on rhythm event sequences rather than vital-sign windows, extending the architecture to a population scale infeasible with in-hospital Holter infrastructure alone.

In all three cases, the narrative layer is more important than in the ICU. A clinician who receives a remote alert lacks the environmental context of a bedside visit, so a structured summary of the triggering observations, the risk driver, and the recommended action is correspondingly more consequential.

### 11.2. Closed-Loop vs. Dashboard Architectures

The fundamental architectural distinction of MedROAD V3 in relation to most CDSS deployed and relative to the published MedROAD V2 [10] is that the EHR is both the source of events and the destination of alerts, eliminating the need for a separate clinical dashboard. On a server that permits it, the loop closes over FHIR in both directions; on OpenEMR 7.0.2 the inbound half is FHIR and the outbound half is that server’s own REST API, for the reason given in Section 8. The published MedROAD V2 solves the same problem in the other direction, being the record system itself and presenting results in its own dashboard and mobile interface. Both approaches satisfy the evidence of Bright et al. [16] and Kawamoto et al. [14] that workflow integration predicts CDSS effectiveness; they differ in what they ask of a site. V2 asks it to adopt a new record system, V3 asks it to accept writes into the one it already runs, which is the lower barrier where an incumbent EHR exists and the harder engineering problem, as Section 11.4 shows. By surfacing its output inside the physician’s existing EHR session, such as native FHIR resources where the server accepts them and as a SOAP note in OpenEMR 7.0.2 where it does not, the system minimises the context-switching overhead that contributes to fatigue of alerts and low adoption rates.

### 11.3. Interface Conformance Against a Reference Deployment

FHIR conformance is often treated as following from the specification: a server that advertises a resource type is assumed to serve it in the shape the specification describes. Exercising MedROAD against a running OpenEMR 7.0.2 instance showed that assumption to be unsafe in a specific and consequential way. Of the faults found, none produced an error, an exception, or an empty result that a monitoring layer would notice, each yielding a plausible output computed from the wrong input. Table 27 records them.

With these resolved, the deployment reads 7 of MedROAD’s 21 channels from charted vital signs, both unit conversions firing, and assembles the full 86-element vector from live data. The 12 laboratory channels are not available because this instance has no laboratory interface, not because of any fault on the path.

The methodological point generalises beyond this server. Every fault above was invisible to a set of unit-tests of 163 tests and to every accuracy metric calculated in MIMIC-IV, because a retrospective evaluation of a research extract never exercises the terminology and unit layers on which a live deployment is based. A conformance check run against the named target server, reporting each capability independently, is therefore part of the deliverable rather than a development convenience, and is distributed with this work. We would encourage reporting its output alongside accuracy metrics for any system claiming EHR integration: the two measure different things, and only one of them is commonly reported.

### 11.4. System Limitations

Several limitations of the current system merit discussion. Where relevant, the comparison with the published MedROAD V2 [10] is made explicit to clarify which limitations are newly introduced in V3 and which are inherited.

A degenerate engineered feature. The interaction score labelled SIG is constant across all physiologically possible inputs and does not contribute anything, and any attribution reported against that position is meaningless (Section 4.3.2). It is one of three columns that do not vary in the evaluated matrix; with a fourth duplicating another, 83 of the 86 columns vary and 82 are distinct (Table 7). Discrimination and calibration results are not affected, since a constant cannot influence a prediction, but the feature is not what the manuscript previously described and the error was ours.

The narrative layer is implemented, not validated. Running-time factual grounding and output-contract checks are in place and tested, and the patient identifier is pseudonymised before transmission. The narrative layer is measured over 180 generated outputs in Section 7.3, which reports gate pass rates, the grounding distribution, and driver selection, but no hallucination rate: establishing one needs clinician adjudication of the generated text and belongs to the prospective study. What the measurement chiefly established is that both runtime gates are inert on this cohort, every rejection either gate has made being a false one, so neither has a demonstrated detection rate. The grounding score is in any case lexical and bound-fabricated rather than clinical correctness.

The clinician evaluation reported for the published MedROAD V2 [10] does not fill this gap: Section Evidence Carried over from MedROAD V2, and Its Limits sets out which of its findings overlap, and the two that overlap are unfavourable to V3.

Comparators. The rule-based scores are the instruments a site would be replacing, not the strongest available models for this cohort. The same-split single-model baselines are reported along with the ensemble in Table 5, but published MedROAD V2 has not been scored on these held-out patients, which limits what the comparison establishes about the gain from V2 to V3 specifically (Section 9.6).

Alert burden against the baselines is reported per window. The figures in Table 17 count windows above threshold rather than clinical episodes, and assume a fully populated shift, so they are upper bounds. The episode-level policy of Section Episode-Level Alerting has been replayed in the holdout windows and the result is shown in Table 16, but it has not been replayed in NEWS or MEWS, so there is no consolidated comparison against the rule-based scores.

Terminology and unit conformance on ingestion. Two classes of silent failure in the live FHIR read path, neither of which produces an error, are set out in Section 11.3. On the terminology side, the LOINC code acts as an internal key in the MIMIC-IV extraction, so an incorrect code is consistent on both sides of the mapping and leaves every result reported here unaffected; on the live path the same mismatch produces an unobserved channel rather than a diagnostic. Therefore, every code in Table 6 was verified individually against its term page, and deployments should repeat that check against their own LOINC release. On the unit side, the normalisation ranges are in MIMIC-IV’s native US conventional units, while SI units are standard in Canada and much of Europe. A conformant server sending creatinine as 88 µmol/L, read 88 mg/dL, pulls to the top of the range and presents a normal result as extreme renal failure; magnesium is worse, as 0.85 mmol/L read 0.85 mg/dL stays within the valid range while being wrong by a factor of 2.4 and thus produces no observable symptom at all. Ingestion converts recognised units and treats an uninterpretable unit as a missing observation, since missingness is already represented in the feature vector, whereas a mis-scaled value corrupts the inference without trace. The conversions are not exhaustive and a deployment should confirm that the units of its source system are among them.

Event delivery against OpenEMR. The Subscription-driven push path of Section 3 cannot be exercised against OpenEMR 7.0.2, which does not implement the Subscription resource. The deployment described here, therefore, uses the polling change feed, and its end-to-end latency is bounded below by the poll interval rather than by delivery. The push path is implemented and used where a server supports it, but the claim that MedROAD removes the batch-ETL latency floor should be read as holding for Subscription-capable servers only. Against OpenEMR the floor is the poll interval, a configurable quantity on the order of tens of seconds, which is of the same order as the 30-s update interval the published MedROAD V2 reports; on this server the architecture’s latency advantage is therefore not demonstrated.

The authorisation model in deployment. MedROAD authenticates as an unattended backend service, which in SMART-on-FHIR terms calls for system/ scopes, the construct intended for exactly that role. OpenEMR 7.0.2 honours system/ scopes only on the client-credentials grant, and authenticates that grant by signed JWT assertion against a registered JSON Web Key Set rather than by client secret. A system/ scope requested in the resource-owner password grant is issued in the token response and then refused at every FHIR endpoint, with a bare 401 and an empty body. The deployment reported here therefore runs under a service account on the password grant with user/ scopes, which is functionally adequate but ties the service to a named user and inherits that user’s access-control list. Sites requiring a genuine service identity must provide a set of accessible keys. The distinction matters for audit, since the actions taken by the service are attributed to the service account.

Write-back is bounded by server capability. None of the four resource types the client emits can be written to OpenEMR 7.0.2, and the obstruction differs by type: three are not among the resource types its CapabilityStatement advertises, and DocumentReference is advertised but carries no write scope (Section 8). Three conditions must hold independently before a write succeeds: the type must be advertised, it must expose a create interaction, and a write scope must be obtainable for it—and they fail separately, so a deployment that checks only the CapabilityStatement, which is the natural thing to check, will conclude that write-back is available when it is not. The interface conformance script distributed with this work checks all three and reports which one blocked each type. The closed-loop claim must therefore be read as conditional on the target server; over FHIR that condition is not satisfied by the reference deployment. The implementation is unchanged, and a server providing the types, the create interactions and the write scopes accepts all four without modification.

The write path that does work is vendor-specific. OpenEMR’s standard REST API, scoped separately under the api:oemr family, does offer clinical write scopes, and MedROAD writes the alert and its narrative there as a SOAP note on the encounter (Section 8.1). That path has been exercised end to end against a live OpenEMR 7.0.2 instance, so the closed loop is achievable on the reference deployment, and the limitation above is a property of OpenEMR’s FHIR surface rather than of the deployment as a whole. The two paths are not equivalent and are not presented as such. The FHIR path travels to any conformant server unchanged; the standard-API path writes OpenEMR’s own records rather than FHIR resources, and a deployment using it is not standards-integrated in the sense the architecture claims. Of the four types emitted, only the narrative destination is confirmed routed in this release; scopes exist for the other three whose creation routes we have not verified, and a scope without a route is not usable. The probe should be run against any target server before relying on any of them.

Outcome definition. Deterioration is defined by a composite of vasopressor initiation, invasive ventilation, and ICU death, assembled from MIMIC-IV event tables. Vasopressor initiation is derived from prescription orders rather than administration records, and ventilator initiation is inferred from the first charted ventilator setting, so both event times are later and noisier than the underlying clinical events. The composite also omits rapid response activation and cardiac arrest, which MIMIC-IV does not record in recoverable form. A deployment site with medication administration records would support a sharper label. The blank window, set to six hours for every result reported here and discussed in Section 9.3, needs to be re-examined in any unit with a different admission profile, and in this cohort it is the single most consequential parameter in the label.

Remote sensor validation. Section 3.6 RPM architecture has not been validated in real wearable streams, and its feature mapping and window reconfiguration are design proposals rather than empirical results. Prospective validation on wearable data, in post-discharge heart failure or diabetic cohorts, is a prerequisite for clinical deployment in the context of telemedicine.

No external or prospective validation. All results in this article derive from MIMIC-IV, a single academic medical centre in the United States (Beth Israel Deaconess Medical Centre, Boston). No external cohort was scored, no local cohort was scored, and no prospective deployment was conducted. The locked partitions are internal: grouped by patient, which removes the leakage that would otherwise inflate them, but drawn from the same institution, era, documentation practice, and case mix as the training data. What the reported AUROC of 0.816 establishes is the internal validity of one site’s historical records, and nothing beyond it. This matters more than a conventional single-site caveat because Section 5.3 shows an operating point that fails outright between cohorts while discrimination and calibration remain intact: a system whose threshold does not transfer should not be assumed to have discrimination that does. Three steps separate this work from a validated system, in increasing order of cost. Score the existing models unchanged on an external cardiac ICU cohort, reporting discrimination and calibration before any recalibration, which measures how much transfers; then apply the site-specific recalibration of Section 6.3 and re-derive the threshold locally, which measures how much of any gap recalibration closes; then the prospective study at the SERVIER Virtual Cardiac Centre. Only the third is currently planned, and we regard the first as the more informative next experiment and the one to run before claiming generalisation at all. Federated training across institutions [51,52] addresses a different problem, training without data movement, and is not a substitute for external validation.

A substantial part of the discrimination is not physiological. Table 14 shows that the twenty-nine care-process columns reach AUROC 0.767 with every physiological measurement withheld, which is 85% of the full model’s discrimination above chance, and that removing them costs 0.007. The reported figure should therefore not be read as a measurement of how well physiological deterioration is detected. Whether the care-process channel reflects genuine severity, which it must in part, or the emerging concern of the bedside team, which would make the score partly a report of a decision already taken, cannot be separated in these data (Section 7.6). A prospective study should be conducted in the physiology-only configuration, where the claim under test is unambiguous.

Distributional shift. Deployment at a site whose demographics, documentation practices, and standard of care differ from MIMIC-IV’s is an expected source of degradation, and it cannot be quantified before prospective data collection begins. The online recalibration pipeline addresses the probability-calibration dimension of this shift, but not changes in the conditional distribution P(Y∣x).

Missingness imputation. Laboratory values missing beyond $L_{j}$ are set to the midpoint of their clipping range, not to a population statistic, unlike V2 [10]. This may introduce systematic bias in patient populations whose lab distributions differ from the training set [63,64]. The two-stage carry-forward imputation introduced in V3 (Section 4.3) reduces but does not eliminate this risk.

LLM reliability. The Claude API narrative generation component is new in V3 and does not have an analogue in the published MedROAD V2 for real-time alerting. It inherits all known limitations of large language models, including potential for hallucination and demographic bias [43]. The mitigations of Section 7.11 bound these risks rather than eliminating them, and the bound is weaker than it may appear. The four that operate are implemented; the fifth, retrieval verification, is not. The grounding and contract gates are measured in Section 7.3 and every rejection either gate has made on the generated output has been a false one, so neither has a demonstrated detection rate. The clinical coherence score is not evaluated in this paper at all. A prospective evaluation of narrative accuracy against expert clinical assessment is planned as part of the SERVIER deployment study.

SHAP limited to XGBoost. The SHAP TreeExplainer provides exact Shapley values for $f_{\mathrm{XGB}}$ only, while the published MedROAD V2 [10] additionally computed the DeepSHAP attributions for its neural components. The LSTM and Transformer contributions to the meta-learner output are not individually explained. The DeepSHAP extension to neural models is planned for a subsequent release.

Security surface. The V3 FHIR Subscription and Kafka ingestion pipeline introduces a networked attack surface that a system acting as its own record store does not have. The signature of The HMAC-SHA256 webhook addresses the most immediate vector, and the prompt does not carry the free-text field, which gives the structural immunity to prompt injection described in Section 7.13 rather than any input sanitization. A formal threat model and penetration test are required prior to clinical production deployment [79].

### 11.5. Future Work

MedROAD V3 moves from an architecture that owns its own record and interface, as the published MedROAD V2 [10] does, towards one that integrates into a third-party EHR: over FHIR where a server allows writes, and over OpenEMR’s standard REST API on the reference deployment, which does. Closing the loop over FHIR awaits a server that accepts the write-back resources. The following directions define the roadmap towards a prospectively validated, multi-site SaMD deployment:

1. Remote patient monitoring pilot. Deployment of the RPM-configured MedROAD V3 pipeline (Section 3.6) on a post-discharge heart failure cohort, comparing alert precision and readmission rates against standard RPM thresholds reviewed by nurses. This would constitute the first prospective validation of the sensor-agnostic FHIR streaming approach in a telemedicine context.
2. Prospective clinical validation at SERVIER. A non-inferiority study comparing MedROAD V3-assisted care to standard NEWS-based monitoring at the SERVIER Virtual Cardiac Centre, with primary endpoints of rapid response activation rate, false alert rate, and time-to-intervention for cardiac deterioration events.
3. Federated learning. Simulated multi-site federated training across partitioned MIMIC-IV as a precursor to real multi-institution deployment, using the FedAvg protocol [50].
4. DeepSHAP extension. Per-inference SHAP values for the LSTM and Transformer components via DeepSHAP, allowing sentence S2 of the narrative to attribute risk to temporal patterns in addition to cross-sectional feature values.
5. Cardiac imaging integration. Incorporation of cardiac motion estimation work by the SERVIER Virtual Cardiac Centre group echocardiographic biomarkers (EF, wall motion score, deformation indices) as additional input features, extending the feature vector beyond the current vital-sign and laboratory domain.
6. Health Canada pre-submission consultation. Formal SaMD pre-submission meeting with Health Canada to establish the device class against a submitted intended-use statement, covering the proposed clinical investigation protocol, risk management documentation according to ISO 14971 and the software lifecycle process according to IEC 62304.
7. V4 architecture scoping. Based on the prospective deployment findings, a V4 architecture would integrate continuous waveform data (ECG, arterial line) through the FHIR Observation streaming profile, adding a branch of the temporal convolutional network to the ensemble for detection of sub-minute arrhythmias.

## 12. Conclusions

This paper has presented MedROAD V3, a real-time FHIR streaming architecture for cardiac clinical decision support, with a write-back layer that closes the loop in two ways: as portable FHIR resources, which the reference deployment cannot accept, and as a SOAP note over OpenEMR’s standard REST API, which it can, at the cost of being vendor-specific. Building directly on the ML foundation established in the published MedROAD V2 [10], MedROAD V3 changes where that foundation runs: event-driven rather than resampled ingestion, calibrated rather than uncalibrated probabilities, and integration into a third-party EHR rather than into the system’s own record (Section What V3 Changes, and What It Does Not).

The five-layer architecture achieves an inference path measured at 3.1 ms at the median, against the 65 ms the published MedROAD V2 allows for ensemble inference and SHAP attribution together. The vector of features is expanded from 72 elements to 86, adding four statistics per vital sign, serial deltas of biomarkers, characteristics of body-weight trend, physiological interaction scores, and metadata encoding documentation timing and intensity of care. The expected calibration error of the ensemble in a holdout cardiac ICU test cohort is 0.001 (Table 5), although Section 6 shows that most of that calibration comes from averaging three models rather than any post-hoc stage, that the isotonic step contributes a real but modest further gain, and that the temperature stage is inert under this training regime. At a similar sensitivity, the calibrated ensemble raises between 39% and 64% fewer alerts than NEWS or MEWS calculated in the same cohort, while discriminating substantially better (AUROC 0.826 vs. 0.590 and 0.578 in patients in the holdout of Table 17; 0.816 in the larger ablation split).

Conditional invocation of the Anthropic Claude API, gated on a calibrated ensemble risk threshold and filtered by a runtime factual-grounding check and an output contract, means that AI-generated clinical narratives are surfaced to physicians only when the deterioration signal is strong, substantially reducing alert frequency relative to the published MedROAD V2’s on-demand summarisation model. Outputs are written back to the patient chart: on a FHIR-capable server as native Flag, CommunicationRequest, DocumentReference, and ServiceRequest resources, and on the reference OpenEMR deployment as a SOAP note written over that server’s own REST API, embedding MedROAD V3 within the clinician’s existing workflow without context-switching.

The formal classification according to Health Canada’s SaMD guidelines, which Section 10 argues is genuinely unsettled rather than determined, and the planned prospective non-inferiority study at the SERVIER Virtual Cardiac Centre together define the path from the current system to a clinically validated medical device. The sensor-agnostic design of the FHIR event source layer further positions MedROAD V3 as a general-purpose multi-modal sensor fusion framework extensible to remote monitoring contexts—post-discharge cardiac surveillance, continuous glucose monitoring, ambulatory arrhythmia detection—without modification to the core streaming or inference architecture, subject to the validation limits of Section 3.6.

## Figures and Tables

**Table 1:** Architectural Comparison: the published MedROAD V2 [10] vs. MedROAD V3.

Dimension	MedROAD V2	This Extension
Ensemble	GB, isolation forest, autoencoder, LSTM	XGBoost, LSTM, Transformer + meta-learner
Data ingestion	15-min resampling, 24-h history	FHIR event-driven, 5-min windows
Inference path	65 ms (estimated)	3.1 ms (measured)
Feature dimension	72	86
Calibration	Not reported	Platt/temp./isotonic + online recal.
Clinician interface	Own dashboard, mobile web	Third-party EHR (write-back)
Alert destination	Own record, pager, in-basket	Third-party chart ^†^
Narrative pipeline	On-demand	Threshold-gated, FGS-filtered
Regulatory framing	Not addressed	Preliminary Health Canada SaMD analysis
Evaluation	All adult ICU, temporal split, admission level	Cardiac ICU, grouped split, window level

The two evaluations are not comparable and the table makes no performance claim: V2 reports AUROC 0.87 at admission level on all adult ICU admissions, V3 reports 0.816 at window level on a cardiac cohort. ^†^ Two paths. The FHIR path is portable but writable only on a server that advertises these resource types, exposes a create interaction for them and offers a corresponding write scope; OpenEMR 7.0.2 satisfies none of the three, so that path is unexercised here. The deployed path uses OpenEMR’s standard REST API, which does accept the write, at the cost of being vendor-specific (Section 8).

**Table 2:** FHIR Subscription Criteria and Kafka Topic Assignments.

Resource	Criteria	Topic	ICU Rate
Observation	category = vital-signs	patient.vitals	∼4/min/pt
Observation	category = laboratory	patient.labs	∼2/h/pt
MedicationRequest	status = active	patient.medications	∼1/h/pt
Encounter	status = in-progress	patient.encounters	Admission/DC
Condition	category = encounter-diagnosis	patient.conditions	∼1/day/pt

**Table 3:** Mapping of ICU monitoring channels to RPM wearable sensor analogues. Bold entries indicate direct substitution into an existing element of the 86-element feature vector; italics indicate approximate functional equivalents. The glucose, arterial MAP and ventilator-pressure rows are ICU channels for which the deployed vector has no element, which is why the five-device kit of Table 4 performs no better than the three-device kit.

ICU Channel	FHIR LOINC	RPM Wearable Analogue
SpO_2_	59408-5	**SpO_2_ (pulse oximeter)**
Heart rate	8867-4	**Heart rate (wrist PPG)**
Systolic BP	8480-6	**Systolic BP (home cuff)**
Weight	29463-7	**Daily weight (smart scale)**
Glucose (serum)	2345-7	*Interstitial glucose (CGM)*
Temperature	8310-5	*Skin temperature (wearable patch)*
Respiratory rate	9279-1	*Breathing rate (accelerometer)*
MAP (arterial)	8478-0	*MAP estimate (oscillometric cuff)*
Troponin I (serial)	10839-9	*Not available—omit or zero-impute*
Ventilator pressure	76248-8	*Not applicable—replace with AHI*

**Table 4:** Retrospective degradation of MIMIC-IV windows to remote monitoring kits, on the reported cohort. All rows are scored by a logistic model on the degraded representation, not by the ensemble, so the reference row is not comparable with the ensemble AUROC reported elsewhere. Channels with no wearable analogue are removed and observable channels are coarsened to their device cadence; the scorer is recalibrated on each degraded representation. Patient-grouped splits throughout, with stratified bootstrap percentile intervals over 2000 replicates.

Deployment	Features	Window	AUROC [95% CI]	ECE	ΔAUROC
ICU (reference, logistic)	86/86	5 min	0.759 [0.756, 0.762]	0.002	—
HF kit (3 devices)	39/86	24 h	0.724 [0.721, 0.728]	0.002	−0.034
Full RPM (5 devices)	39/86	24 h	0.724 [0.721, 0.728]	0.002	−0.034

The two kit rows are equal by construction rather than by measurement: they reduce to the same 39 feature columns, because the additional devices publish channels this feature vector has no element for. ΔAUROC is computed on the unrounded values and so differs by 0.001 from the difference of the rounded column.

**Table 5:** Discrimination and calibration on held-out patients of the reported cohort (435,307 windows, 19,285 positive, event rate 4.4%). Splits are grouped by patient, so no individual contributes to both training and evaluation. Intervals are stratified bootstrap percentile intervals over 2000 replicates. The *p*-value is DeLong’s test for the AUROC difference against the full ensemble on the same windows.

Variant	AUROC [95% CI]	ECE [95% CI]	Brier	*p* vs. Full
XGBoost	0.805 [0.802, 0.808]	0.004 [0.004, 0.005]	0.0386	<0.001
LSTM	0.805 [0.802, 0.808]	0.334 [0.333, 0.335]	0.1786	<0.001
Transformer	0.810 [0.807, 0.813]	0.372 [0.371, 0.372]	0.2027	<0.001
Logistic (same split)	0.758 [0.754, 0.761]	0.002 [0.002, 0.003]	0.0388	<0.001
Ensemble without Transformer	0.808 [0.804, 0.811]	0.001 [0.001, 0.002]	0.0375	<0.001
Ensemble without LSTM	0.814 [0.811, 0.817]	0.002 [0.001, 0.002]	0.0375	<0.001
Ensemble without XGBoost	0.814 [0.811, 0.817]	0.002 [0.001, 0.002]	0.0378	<0.001
Ensemble (full)	0.816 [0.813, 0.819]	0.001 [0.001, 0.002]	0.0373	—

Both the intervals and the *p*-value take a five-minute window as the sampling unit, which it is not: these windows come from 5049 patients. Both therefore overstate the evidence, the *p*-values by an unknown and potentially large factor, and neither column should be read as architecture-level inference. Table 13 gives the patient-clustered version of both. DeLong’s test is at least paired on the same windows, so it resolves differences the marginal intervals leave overlapping, but pairing addresses correlation between the two variants and not correlation between windows of one patient.

**Table 6:** Vital Sign and Laboratory LOINC Codes.

Parameter	LOINC Code	Unit
Vital Signs (9)		
Heart rate	8867-4	/min
Systolic BP	8480-6	mmHg
Diastolic BP	8462-4	mmHg
SpO_2_	59408-5	%
Respiratory rate	9279-1	/min
Temperature	8310-5	°C
Glasgow Coma Score	9269-2	score
Pain score (0–10)	72514-3	score
Body weight	29463-7	kg
Laboratory Values (12)		
Cardiac troponin	10839-9	ng/mL
NT-proBNP	33762-6	pg/mL
Creatinine	2160-0	mg/dL
Potassium	2823-3	mmol/L
Sodium	2951-2	mmol/L
Bicarbonate	1963-8	mmol/L
pCO_2_ (arterial)	2019-8	mmHg
Blood pH	11558-4	–
Lactate (blood)	32693-4	mmol/L
Hematocrit	4544-3	%
WBC	6690-2	K/μL
Magnesium	2601-3	mg/dL

Codes verified individually against their LOINC term pages (release 2.82). The unit column gives the unit the feature normalisation assumes, which is MIMIC-IV’s native unit and in one case not the unit the LOINC term is defined in: magnesium is handled in mg/dL although 2601-3 is a molar term. Observations arriving in another valid unit are converted on ingestion, and an uninterpretable unit is treated as a missing observation rather than normalised as though it were canonical (Section 11.4).

**Table 7:** What actually varied in the matrix behind every reported result. Index ranges refer to Table 10.

Column	Status in the Evaluated Matrix	Count
All columns in the vector		86
sig (82)	Identically 1.0; saturates for every admissible input	−1
meta_med_burden (53)	Identically 0; no prescription records in the extract	−1
meta_iv_drip (54)	Identically 0; same cause	−1
Varying columns		83
meta_vital_recency (52)	Assigned the value of meta_doc_lag (51)	−1
Distinct varying columns		82

**Table 8:** Laboratory feature missingness rates and imputation parameters on the MIMIC-IV cardiac cohort. No percentile of an admission-level cohort enters these parameters; the bounds are clinically chosen, as Section 4.3.3 sets out.

Parameter	Missing (%)	Median	IQR	$L_{j}$ (h)
Troponin I	6.2	0.08	0.03–0.41	48
NT-proBNP	18.7	312	98–1 180	48
Creatinine	4.1	1.0	0.8–1.4	24
Potassium	3.8	4.0	3.6–4.4	24
Sodium	3.5	138	136–141	24
Bicarbonate	8.9	24	22–27	8
pCO_2_	61.2	40	35–46	8
Arterial pH	62.4	7.40	7.36–7.44	8
Lactate	54.8	1.4	1.0–2.1	8
Hematocrit	5.3	33	29–38	24
WBC	5.1	10.2	7.8–13.6	24
Magnesium	22.3	1.9	1.7–2.1	24

**Table 9:** Metadata Feature Specification ($x_{\mathrm{meta}}\in R^{8}$).

Feature	Range	Rationale
Group 1: Data Coverage		
$x_{\mathrm{vital}\_\mathrm{cov}}$	[0,1]	Fraction of 9 vital LOINC types charted in current window
$x_{\mathrm{lab}\_\mathrm{cov}}$	[0,1]	Fraction of 12 lab types available within 24 h
$x_{\mathrm{abg}\_\mathrm{present}}$	{0,1}	Binary: ABG panel (pH, pCO_2_, lactate) drawn within 4 h
Group 2: Documentation Timing		
$x_{\mathrm{doc}\_\mathrm{lag}}$	[0,1]	Normalised time since most recent nursing documentation
$x_{\mathrm{vital}\_\mathrm{recency}}$	[0,1]	Normalised age of most recent vital-sign observation
Group 3: Care Intensity		
$x_{\mathrm{med}\_\mathrm{burden}}$	[0,1]	Active medication count, capped and normalised
$x_{\mathrm{iv}\_\mathrm{drip}}$	{0,1}	Binary: any continuous IV infusion active
Group 4: Patient Temporal Context		
$x_{\mathrm{icu}\_\mathrm{los}}$	[0,1]	ICU length of stay in days, log-normalised

**Table 10:** Definitive feature schema. Index ranges are positions in x as assembled by the feature builder.

Index	Block	*n*	Contents
0–35	Vital statistics	36	Mean, standard deviation, minimum and rate of change for each of 9 vital signs
36–47	Laboratory values	12	Most recent value within the look-back $L_{j}$, min-max scaled to [0,1] over a clipping range
48–55	Metadata	8	Coverage (2), ABG present, documentation lag, vital recency, medication burden, IV infusion, ICU length of stay
56–64	Vital missingness	9	One indicator per vital channel
65–76	Laboratory missingness	12	One indicator per laboratory channel
77–79	Serial laboratory deltas	3	Δtroponin, ΔNT-proBNP, Δlactate
80–81	Weight trend	2	24-h and 72-h weight change
82–83	Interaction scores	2	SIG (see note), BCR = NT-proBNP / creatinine
84–85	Temporal context	2	sin and cos of hour of day
Totals D=36+12+8+9+12+3+2+2+2=86			

What each learner receives. XGBoost: $x\in R^{86}$, one window. LSTM and Transformer: $X\in R^{T\times 86}$, the same 86 features over *T* consecutive windows. Meta-learner: $\left({\hat{p}}_{\mathrm{XGB}},{\hat{p}}_{\mathrm{LSTM}},{\hat{p}}_{\mathrm{TF}}\right)\in R^{3}$, the calibrated base probabilities only. SHAP: TreeExplainer over all 86 positions of $f_{\mathrm{XGB}}$. Note on index 82. The interaction score labelled SIG is degenerate in the implementation evaluated here and contributes no information; see Section 4.3.2. It is one of three columns that do not vary in the evaluated matrix, and index 52 duplicates index 51, so of these 86 positions 83 vary and 82 are distinct. Table 7 gives the reconciliation. The grouping of Equation (7) differs only in bookkeeping: its $|x_{\mathrm{lab}}|=17$ counts the 12 laboratory values together with the 3 serial deltas and the 2 interaction scores.

**Table 11:** SHAP attribution stability against background sample size, over ten redraws at each size on the reported cohort. Jaccard is the mean overlap of the top-five feature set between redraws; the adjacent column expresses it as features in common out of five. CV is the coefficient of variation of the attribution, over all features and over the eight metadata features alone.

Background	Top-5 Jaccard	of 5	Spearman	CV All	CV Metadata
25	0.776	4.37	0.857	0.333	0.107
50	0.825	4.52	0.900	0.293	0.078
100	0.869	4.65	0.938	0.229	0.049
200	0.882	4.69	0.931	0.280	0.062
500	0.870	4.65	0.931	0.340	0.045

**Table 12:** Target-band AUROC across four independent runs of the same experiment on the same data and split. Variation between columns is retraining alone. The margin rows are differences within a run, which are paired on shared base-model fits.

Variant	Run 1	Run 2	Run 3	Run 4	Range
XGBoost	0.801	0.801	0.801	0.801	0.000
LSTM	0.799	0.805	0.803	0.804	0.006
Transformer	0.806	0.811	0.804	0.812	0.008
Logistic (source-fitted)	0.756	0.756	0.756	0.756	0.000
Ensemble without Transformer	0.802	0.807	0.805	0.806	0.005
Ensemble without LSTM	0.809	0.814	0.807	0.814	0.007
Ensemble without XGBoost	0.810	0.816	0.810	0.814	0.006
Ensemble (full)	0.812	0.817	0.812	0.815	0.005
Leave-one-out margin, full ensemble minus the reduced variant					
without Transformer	0.010	0.010	0.007	0.009	0.003
without LSTM	0.003	0.003	0.005	0.001	0.004
without XGBoost	0.002	0.001	0.002	0.001	0.001

**Table 13:** Patient-clustered inference for Table 5. The same held-out predictions under two assumptions about the sampling unit. The left interval and DeLong’s test treat each five-minute window as independent; the right interval and the clustered *p*-value resample patients, carrying all of a patient’s windows together.

Variant	Window-Unit CI	Patient-Unit CI	Width	*p* DeLong	*p* Clustered
XGBoost	0.802, 0.808	0.793, 0.818	3.9×	<0.001	0.002
LSTM	0.802, 0.808	0.793, 0.817	3.7×	<0.001	0.002
Transformer	0.807, 0.813	0.799, 0.822	3.7×	<0.001	0.002
logistic (same split)	0.754, 0.761	0.744, 0.771	3.6×	<0.001	0.002
ensemble without Transformer	0.804, 0.811	0.796, 0.819	3.7×	<0.001	0.002
ensemble without LSTM	0.811, 0.817	0.802, 0.825	3.7×	<0.001	0.016
ensemble without XGBoost	0.811, 0.817	0.803, 0.826	3.8×	<0.001	0.002
ensemble (full)	0.813, 0.819	0.805, 0.828	3.8×	—	—

Clustered quantities are percentile bootstrap over 500 patient resamples, so the clustered *p*-value cannot fall below 1/500=0.002; every row showing 0.002 is at that floor, meaning no resample reversed the sign of the difference, and is not a measured value of 0.002. The clustered *p*-value inverts the interval on the paired difference against the full ensemble. Windows from one stay share physiology, charting practice and a single outcome, so the window-unit columns understate uncertainty by the width ratio shown.

**Table 14:** Discrimination under removal of the care-process block. Metadata columns record documentation timing, channel coverage and intensity of care; missingness columns record which measurements were not taken. Every subset is refit on the same patient-grouped split and scored on the same held-out patients.

Feature Set	Columns	AUROC [95% CI]	Δ vs. Full	ECE
full	86	0.815 [0.802, 0.827]	—	0.001
no metadata	78	0.815 [0.803, 0.826]	−0.000	0.001
no missingness	65	0.815 [0.801, 0.826]	−0.000	0.001
physiology only	57	0.808 [0.795, 0.821]	−0.007	0.001
care process only	29	0.767 [0.752, 0.782]	−0.048	0.000

Intervals are patient-clustered percentile bootstrap. The care-process-only row has no access to any physiological measurement, so its AUROC bounds how much of this outcome is predictable from how the patient was managed and documented. A drop on removal is not proof of leakage, since documentation intensity also tracks genuine severity, and a small drop is not proof of its absence.

**Table 15:** Operating points derived on held-out scores. The configured constant of 0.72 is shown for comparison; it was never fitted.

Operating Point	τ	Sensitivity	Precision
$F_{1}$ maximum	0.153	0.352	0.279
80% sensitivity	0.037	0.800	0.103
90% sensitivity	0.023	0.900	0.080
95% sensitivity	0.012	0.950	0.068
Former constant	0.720	0.043	—

**Table 16:** Alert burden at matched sensitivity, replaying the episode policy over held-out patients. Window-level counts are what the system as originally evaluated would deliver; episode-level counts are what the consolidation policy delivers from the same predictions.

Quantity	At τ=0.72	At 90% Sensitivity
Threshold	0.720	0.028
Window-level alerts per patient-day	0.14	21.20
Episode-level alerts per patient-day	0.06	2.11
Reduction from consolidation	55.7%	90.1%
Window-level sensitivity	0.043	0.910
Stay-level sensitivity	0.129	0.990

The threshold here differs from the 0.023 of Table 15 because the two are derived on different held-out sets: that table uses the 435,307-window ablation split and this one the 160,748 windows of the saved model’s own split. Measured on 160,748 held-out windows over 3514 stays and 4529 patient-days. Both columns are the same predictions read at different thresholds, so the reduction is not bought by detecting less.

**Table 17:** Discrimination and alert burden at matched sensitivity. Alerts are per patient per 8-h shift, which contains 96 five-minute windows. Comparison at equal sensitivity is what makes the burden figures meaningful.

System	AUROC [95% CI]	@ 70%	@ 80%	@ 90%	*p* vs. V3
NEWS	0.590 [0.586, 0.595]	58.72	71.14	82.82	<0.001
MEWS	0.578 [0.573, 0.582]	61.43	87.92	87.92	<0.001
MedROAD V3	0.826 [0.822, 0.829]	22.00	33.33	50.27	—

Oxygen supplementation is not recorded in this cohort and is scored zero in NEWS, which slightly understates it. MEWS is integer-valued over five channels, so the smallest threshold reaching 80% sensitivity is the same integer as the one reaching 90%; its two right-hand columns are therefore identical by construction rather than by coincidence. The per-shift figures assume a fully populated shift of 96 consecutive five-minute windows. The held-out cohort supplies 160,748 windows over 4529 patient-days, which is 35.5 windows per patient-day rather than the 288 a fully populated day would contain, so these columns are upper bounds on a per-shift count and Table 16, which divides by the patient-days actually observed, is the figure to quote for burden. All three systems are converted identically, so the ratios between them are unaffected.

**Table 18:** Transportability across admission era: trained on MIMIC-IV admissions from 2008–2019 (1,554,445 windows, event rate 0.0441), evaluated on 2020–2022 (192,452 windows, 0.0603). The site is unchanged; this is not external validation.

Variant	AUROC [95% CI]	ECE [95% CI]	Brier
XGBoost ^‡^	0.801 [0.797, 0.805]	0.018 [0.017, 0.019]	0.0523
LSTM	0.804 [0.800, 0.808]	0.281 [0.280, 0.282]	0.1658
Transformer	0.812 [0.808, 0.816]	0.294 [0.293, 0.295]	0.1543
Logistic (source-fitted) ^‡^	0.756 [0.751, 0.761]	0.010 [0.010, 0.011]	0.0515
Ensemble −Transformer	0.806 [0.802, 0.810]	0.001 [0.000, 0.002]	0.0499
Ensemble −LSTM	0.814 [0.810, 0.818]	0.001 [0.001, 0.002]	0.0495
Ensemble −XGBoost	0.814 [0.810, 0.818]	0.002 [0.001, 0.003]	0.0497
Ensemble (full) ^†^	0.815 [0.812, 0.819]	0.000 [0.000, 0.002]	0.0494
Ensemble (source-fitted)	0.817 [0.813, 0.821]	0.019 [0.019, 0.020]	0.0512

One run of four; the intervals are bootstrap intervals within that run and do not cover the variation between runs, which Table 12 reports. ^‡^ Deterministic under the fixed seed: these two rows are identical in all four runs. ^†^ The base models transfer: they are fitted on the source bands and scored on the target. In every ensemble row above the source-fitted one the meta-learner and the isotonic calibrator are refitted out of fold on the target band’s labels, so those rows measure what a site gets after recalibrating locally. The source-fitted row freezes the whole combination layer on the source and applies it unchanged, which is what a site inheriting a trained system actually receives; the difference between the two is the subject of the discussion below. Recalibrating the full ensemble on half the target band and evaluating on the other half gives ECE 0.000 against 0.000 without recalibration, which is unsurprising because that row is already refitted on the target.

**Table 19:** Transferability of MedROAD V2’s language-model evaluation to V3.

V2 Finding	Transfers?	Why
Diagnostic precision 84%/94%	No	V3 produces no differential diagnosis.
Citation accuracy 89%	No	V3 retrieves no literature and emits no citations.
Fabricated citations, 10–30% unmitigated	No	Same reason; this is V2’s largest hallucination category and V3 does not incur it.
Drug dosing errors, 15–25% unmitigated	Yes	V3’s narrative recommends an action and may name an agent, so this category is live.
Guideline specifics, 10–20% unmitigated	Partly	V3 makes no explicit guideline claim, but a recommended action implies one.
Web search cuts hallucination 10%→3%	Not available	V3 performs no retrieval, so it operates in the unmitigated regime for the categories that do transfer.

**Table 20:** Illustrative narrative output for a constructed case (R=0.84), showing the required structure. Not a generated sample.

Input	Value
HR (mean/RoC)	118 /min; +4.2 /min/min
BP (mean)	84/52 mmHg
Lactate	4.8 mmol/L (Δ+1.2 in 4 h)
NT-proBNP	2 840 pg/mL
Top SHAP (ϕ)	BP_sys_roc (+0.41), lactate (+0.38), HR_mean (+0.22)
Medications	Furosemide 40 mg IV, Metoprolol 25 mg PO, Lisinopril 5 mg PO
S1	Haemodynamic profile consistent with low-output state: persistent hypotension (MAP ≈ 63 mmHg), tachycardia with accelerating heart rate, and markedly elevated NT-proBNP indicating ventricular wall stress.
S2	Primary risk drivers by SHAP analysis: declining systolic BP trajectory (ϕ=+0.41), rising lactate now 4.8 mmol/L above critical threshold (ϕ=+0.38), and sustained tachycardia (ϕ=+0.22), collectively consistent with early cardiogenic shock.
S3	Consider urgent reassessment of volume status and haemodynamic monitoring; vasopressor initiation (norepinephrine) may be indicated if MAP < 65 mmHg is confirmed; bedside echocardiography to assess contractility is recommended.
S4 (DDI)	Note: concurrent furosemide and lisinopril with hypotension may exacerbate haemodynamic compromise; consider holding both agents pending haemodynamic stabilisation.
Disclaimer	AI-generated decision support summary—physician review required.

**Table 21:** Write-back over OpenEMR’s standard REST API, as deployed.

SOAP Section	Content
Subjective	A statement that the assessment is automated and uses charted physiology only, so the note cannot be read as implying a history was taken.
Objective	The monitoring window and the ensemble risk score against the calibrated threshold.
Assessment	The risk score, the threshold it exceeded, and the principal model drivers with their SHAP attributions.
Plan	The generated narrative, followed by the provenance statement: machine-generated, physician review required, no order implied or placed, with the authoring system and timestamp.

**Table 22:** FHIR resource mappings for write-back. Implemented and unit-tested; not exercisable on OpenEMR 7.0.2, which offers no write scope for any FHIR clinical resource.

MedROAD Output	FHIR Resource	EHR Visibility
Risk alert (R≥τ)	Flag	Patient banner
Drug interaction warning *	DetectedIssue	Medication list icon
Physician communication	CommunicationRequest	Task list
Claude API narrative	DocumentReference	AI notes section
Recommended order	ServiceRequest	Order entry (draft)
Alert resolved (R<τ)	Flag (inactive)	Banner cleared

* Specified by the design but not emitted by the write-back client in this release; the client builds the other four resource types. Counts of emitted types elsewhere in the paper therefore exclude it.

**Table 23:** Cohort statistics under each outcome definition, MIMIC-IV v3.1. Event-type columns count stays affected, and a stay may appear under more than one type, so they exceed the combined total. The vasopressor list is the five-agent default except in the row marked marginal, which adds phenylephrine and milrinone.

Care Units	Blank	Stays	Vasop.	Vent.	Death	Any	Rate
CCU + CVICU	1 h	25,214	—	11,677	1469	—	—
CCU + CVICU	1 h, marginal	25,214	12,795	11,677	1469	15,678	62.2%
CCU + CVICU	6 h	25,214	4478	2228	1469	6000	23.8%
CCU only	1 h	10,576	2864	1333	1005	3591	34.0%
CCU only	6 h	10,576	1968	546	1005	2500	23.6%

The first row is an incomplete extraction retained because its ventilation count alone establishes the result; its vasopressor pass was not run to completion. The counts here are of stays carrying an event in the extraction. The modelling cohort of Section 9.3 carries 5907 rather than the 6000 in the six-hour row, because window generation emits nothing for a stay with no charted observation in any five-minute interval before its event or discharge, and 93 stays meet that description. The two numbers count different things and neither supersedes the other.

**Table 24:** Per-stage compute latency, measured on windows from the evaluation cohort (NVIDIA GPU, models resident). Each row is a separate timing loop over independently sampled windows. The total row is measured end-to-end rather than summed: it excludes feature assembly and it incurs SHAP attribution only on windows that cross the alert threshold, so it is not the column sum.

Stage	p50 (ms)	p95 (ms)	p99 (ms)	max (ms)
Feature assembly	0.22	0.24	0.34	0.50
XGBoost inference	0.76	0.85	0.94	1.08
LSTM inference	0.28	0.40	0.61	0.87
Transformer inference	0.70	1.06	1.23	1.51
Meta-learner	0.07	0.08	0.17	0.26
SHAP attribution	2.55	3.15	3.75	4.49
Compute path (total)	3.09	3.40	3.72	10.06

**Table 25:** End-to-end latency budget. Compute (Table 24) and the two transport stages of Table 26 are measured; the remaining rows are architectural estimates and are marked as such. Totals inherit that status.

Stage	Mechanism	Latency	Source
Vital charted → delivery	REST-hook, or poll	poll interval	not measured
Webhook receipt → Kafka ack	HTTP POST + produce	3.1 ms	measured
Kafka consumer lag (Faust)	Consumer group poll	∼50 ms	estimated
Compute path	In-process	3.1 ms	measured
Write-back to OpenEMR	REST API POST	33–35 ms	measured ^‡^
Total after delivery		∼90 ms	
Narrative generation	Anthropic API	∼1800 ms	estimated
Total with narrative		∼1890 ms	

**Table 26:** Transport latency measured against the running deployment, over repeated round trips with synthetic payloads. The webhook figure is the time from receipt of a POST to acknowledgement and includes the Kafka produce performed inside the handler; the Kafka row times a direct produce in isolation. Neither includes the EHR detecting a change and dispatching it, which the polling path bounds by the poll interval.

Transport Stage	p50 (ms)	p95 (ms)	p99 (ms)	Max (ms)
Webhook receipt → Kafka ack	3.1	4.8	25.8	26.9
Kafka produce (acks = all)	0.8	1.1	1.2	5.8

All services co-located on one host; a distributed deployment adds network latency to every stage.

**Table 27:** Interface faults found by exercising MedROAD against OpenEMR 7.0.2. Each was silent: the pipeline continued and produced a score.

Server Behaviour	Consequence If Unhandled	Resolution
Vital signs returned as panel Observations (85353-1, 85354-9) with measurements in component	The panel code is unmapped, so the whole resource is discarded and all nine vital channels read as unobserved. The vector is imputed midpoints throughout and a risk score is still produced.	Components treated as first-class measurements, each with its own code, value and unit.
Temperature in °F, weight in pounds	98.6 °F read as 98.6 °C clips to the top of the normalisation range; 172 lb read as 172 kg falls inside it and is simply wrong.	Unit normalisation on ingestion; an uninterpretable unit is treated as a missing observation.
Several LOINC codes on one measurement (US Core pairs 2708-6 with 59408-5 for ${\mathrm{SpO}}_{2}$)	Reading only the first coding makes ingestion depend on the server’s ordering of synonyms.	Every coding resolved; the first that maps determines the channel.
_count not supported, answered with an empty bundle	Identical to the patient having no data. The pipeline reads nothing and reports no fault.	Optional search parameters dropped and the query retried when a result is empty.
Observation search requires patient context	A server-wide change feed returns nothing, indistinguishable from a quiet ward.	The monitored cohort is named explicitly and queried per patient.

## Data Availability

The MIMIC-IV critical care database is publicly available via PhysioNet at https://physionet.org/content/mimiciv/3.1/ subject to completion of the required credentialing and data use agreement. The database was accessed on 12 June 2026. The MedROAD V3 reference implementation, including the FHIR client, feature engineering pipeline, ensemble inference engine, and Docker deployment configuration, is available from the author on reasonable request. No new clinical data were generated for this study.
